# Omegasome-proximal PtdIns(4,5)P_2_ couples F-actin mediated mitoaggregate disassembly with autophagosome formation during mitophagy

**DOI:** 10.1038/s41467-019-08924-5

**Published:** 2019-02-27

**Authors:** Cheng-Wei Hsieh, Wei Yuan Yang

**Affiliations:** 1 Institute of Biological Chemistry, Academia Sinica, Taipei, Taiwan; 2 Institute of Biochemical Sciences, College of Life Sciences, National Taiwan University, Taipei, Taiwan

## Abstract

Cells govern their homeostasis through autophagy by sequestering substrates, ranging from proteins to aggregates and organelles, into autophagosomes for lysosomal degradation. In these processes cells need to coordinate between substrate remodeling and autophagosome formation for efficient engulfment. We found that in Parkin-mediated mitophagy, mitochondria to be turned over first become grape-like mitoaggregates, followed by their disassembly into smaller pieces via the actinomyosin system. At the disassembly step, we observed spatially-associated, synchronous formation of circular F-actin and BATS-labeled autophagy initiation sites near mitochondria, suggesting coordination between substrate downsizing and autophagosome formation during mitophagy. Interestingly, PtdIns(4,5)P_2_, instead of PtdIns(3)P, regulates this mitophagy-associated formation of circular F-actin and BATS-sites. Selective depletion of PtdIns(4,5)P_2_ near omegasomes, the endoplasmic reticulum (ER) subdomains involved in autophagosome formation, impaired mitoaggregate disassembly. Our findings demonstrate the presence of a pool of PtdIns(4,5)P_2_ adjacent to omegasomes, and that they coordinate mitoaggregate disassembly with autophagy initiation during Parkin-mediated mitophagy.

## Abstract (web summary)

Autophagic cells coordinate substrate remodeling with sequestration during autophagosome formation. Here, the authors show that during Parkin-mediated mitophagy, mitochondria are disassembled into progressively smaller aggregates near autophagy initiation sites in a PtdIns(4,5)P_2_-dependent manner.

## Introduction

Mitochondrial autophagy (mitophagy) is an organelle quality control pathway that sequesters damaged mitochondria into autophagosomes for lysosomal removal^1,2^. Defective mitochondria form clusters of mitochondrial aggregates (hereafter mitoaggregates) and can accumulate at the perinuclear region, a signature phenomenon reported in Parkinson’s disease model systems^3^. Similar to the generation of protein aggresomes, mitoaggregate formation is considered a protective mechanism that clusters defective materials for the reduction of cellular toxicity. This process is driven by (1) Pink1/Parkin-mediated ubiquitination of damaged mitochondria and subsequent accumulation of the ubiquitin (Ub) receptor p62 (also known as SQSTM1) that help cluster impaired mitochondria^4,5^, and (2) Trafficking of damaged mitochondria to the perinuclear region^6,7^. The apparent size mismatch between autophagosomes^8^ (typically 0.5–1.5 μm in mammalian cells) and mitoaggregates suggests that mitoaggregates will eventually need to be dimensionally remodeled for efficient mitophagy.

A particularly interesting aspect related to mitoaggregate remodeling is its relationship with the autophagy initiation process. Synchronization between mitoaggregate remodeling and mitochondrial targeting by the autophagic machinery can reduce the exposure of free floating dysfunctional mitochondria to the cytoplasm, thereby minimizing cellular stress. It is known that autophagosomes originate from PtdIns(3)P-rich sites on the ER termed omegasomes:^9^ LC3-PE containing autophagosomal membrane grows from these regions, which in turn sequesters substrates into complete autophagosome. Positioning mitoaggregate remodeling events directly at the omegasomes therefore represents one way to seamlessly integrate Parkin-labeled mitochondrial fragments into the autophagy pathway.

Here, we report that in Parkin-mediated mitophagy the mitoaggregates represent clusters of fragmented and swollen mitochondria. These mitoaggregates require disassembly into smaller pieces for productive Parkin-mediated mitophagy; during this process, circular F-actin and BATS-labeled autophagy initiation structures cooperatively form in proximity. The formation of both structures is controlled by the same upstream activator, a pool of PtdIns(4,5)P_2_ that resides near the omegasome. Our results reveal the unrecognized role of phosphoinositides (PtdIns) in coordinating mitoaggregate remodeling with autophagy initiation during Parkin-mediated mitophagy.

## Results

### Mitoaggregate formation and disassembly during mitophagy

**Fig. 1:**
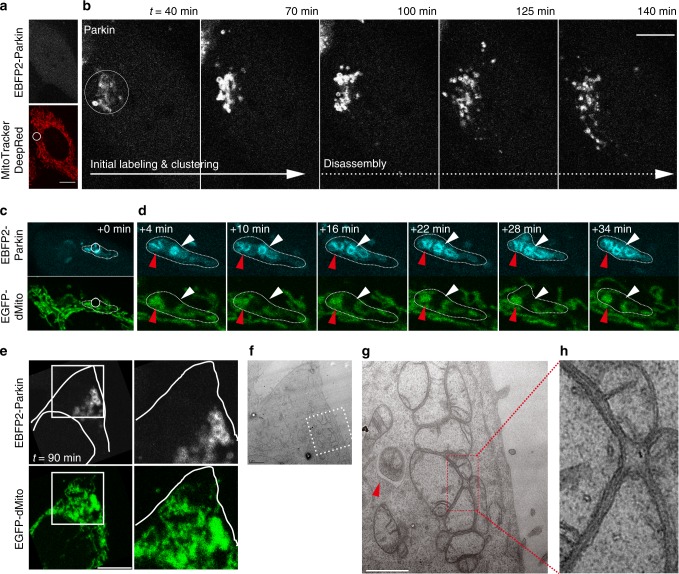
Mitoaggregate formation and disassembly in light-induced Parkin-mediated mitophagy. **a** MitoTracker DeepRed FM-stained HeLa cells expressing EBFP2-Parkin were illuminated with 635 nm (white circle; immediate loss of MitoTracker DeepRed FM fluorescence) to locally activate Parkin-mediated mitophagy. Scale bar: 10 μm. **b** Selected frames from a time-lapse movie (Supplementary Movie 1) on the cellular region undergoing mitophagy in **a** after 635 nm illumination. Upon initial Parkin-labeling (left), mitochondria underwent biphasic morphology changes, including clustering (middle) and disassembly (right). **c**, **d** Analysis of mitoaggregate connectivity by FRAP. **c** MitoTracker DeepRed FM-stained HeLa cells co-expressing EBFP2-Parkin, EGFP-dMito, and KillerRed-dMito were illuminated with 559 nm to generate Parkin-labeled mitoaggregates (white-dotted region). EGFP-dMito within a small portion of clustered mitoaggregates were photobleached by 488-nm light (white circle). **d** Magnified view of clustered Parkin-labeled mitoaggregates. EGFP-dMito within the 488-nm illuminated regions (white arrow) were photobleached and monitored for recovery over a course of 30 min (none observed). A non-photobleached regions (red arrow) served as an internal controls. **e–g** CLEM analysis of Parkin-labeled mitoaggregates. **e** MitoTracker DeepRed FM-stained HeLa cells co-expressing EBFP2-Parkin and EGFP-dMito were illuminated with 635 nm to generate Parkin-labeled mitoaggregates. The cells were fixed 90 min after illumination and analyzed by electron microscopy. Scale bar: 10 μm. **f** TEM image correlating to the region of interest under fluorescence microscopy (white square region in **e**). **g** Magnified view of the white-dotted square region in **f**. Grape-like mitoaggregates were larger than mature autophagosomes (red arrow). Scale bar: 1 μm. **h** The expanded view shows that there are high electron densities at the interface of mitoaggregates

Using a photosensitizer^10^ to selectively mediate the impairment of a fraction of cellular mitochondria (Fig. 1a), we observed that damaged mitochondria underwent mitoaggregate formation and disassembly within a time span of 3 h (Fig. 1b; Supplementary Movie 1): first they clustered into mitoaggregates after Parkin recruitments (40–50 min after damage), followed by their disassembly into smaller fragments (90–100 min after damage). We then utilized FRAP to probe the status of Parkin-labeled mitoaggregates in HeLa cells expressing EBFP2-Parkin, EGFP-dMito (a mito-matrix-targeting EGFP), and KillerRed-dMito (another photosensitizer for mitophagy initiation). Compared to the control where EGFP-dMito can exchange between interconnected mitochondria (Supplementary Fig. 1a), EGFP-dMito was unable to freely exchange within mitoaggregates (Fig. 1c, d; white arrow indicates the photobleached region). This demonstrated that the mitoaggregates were not composed of an entangled tubular mitochondrion. Correlative light and electron microscopy (CLEM) revealed that mitoaggregates represent clusters of fragmented and swollen mitochondria (Fig. 1e–g and Supplementary Fig. 1b, c). This indicated that damaged mitochondria underwent mitochondrial fission as well as clustering to form mitoaggregates, followed by their disassembly into smaller fragments for turnover. High electron densities found at the interfaces between individual mitochondrial fragments in the mitoaggregates (Fig. 1 h) support the presence of tethering factors (such as Ub and p62) that help mitoaggregate clustering. In support of this, p62-deficient HeLa cells exhibited defective formation of mitoaggregates (Supplementary Fig. 1d). As shown in Fig. 1g, the dimensions of grape-like mitoaggregates are much larger than those of typical autophagosomes (Fig. 1g; red arrow); thus, the disassembly of mitoaggregates into smaller pieces appears to be necessary for their efficient engulfment into autophagosomes. We therefore sought to determine how cells regulate mitoaggregate disassembly.

### Circular F-actin structures during mitoaggregate disassembly

**Fig. 2:**
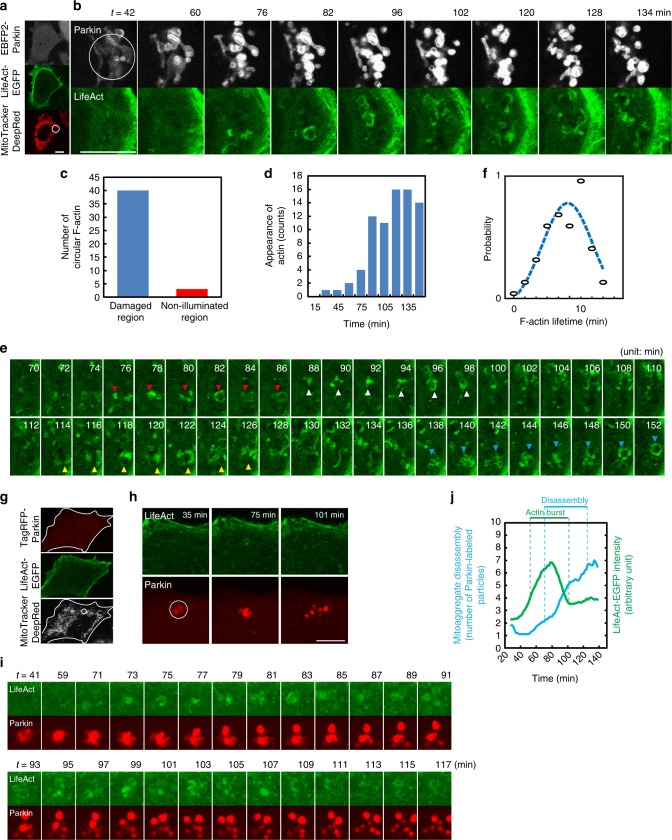
Circular F-actin formation during mitoaggregate disassembly. **a** MitoTracker DeepRed FM-stained HeLa cells co-expressing EBFP2-Parkin and LifeAct-EGFP were illuminated with 635 nm (white circle; immediate loss of MitoTracker DeepRed FM fluorescence) to locally activate Parkin-mediated mitophagy. **b** Selected frames from a time-lapse movie (Supplementary Movie 2) on the cellular region undergoing mitophagy in **a** after 635 nm illumination. **c** Measurements of total circular F-actin structures in the damaged and non-illuminated region (as shown in Supplementary Fig. 3a) of the cell in **a**. Circular F-actin structures preferentially occurred in the illuminated region where Parkin-mediated mitophagy was initiated. **d** Time-dependent formation of circular F-actin structures in the damaged region of the cell in **a**. Circular F-actin structures primarily occurred 75 min after illumination (during the mitoaggregate disassembly phase). **e** Time-lapse analysis of the dynamics of circular F-actin. Newly formed circular F-actin structures are indicated by colored arrows. **f** Lifetime of circular F-actin structures (calculated from *n* = 235 events). **g** A small portion of the mitochondria were damaged in a HeLa cell. **h** Magnified view of the cell in **g**. Representative images were selected to indicate the impaired mitochondria undergoing initial Parkin labeling, clustering, and disassembly. **i** Selected frames from a time-lapse movie (Supplementary Movie 3) of the cell in **g**. Several circular F-actin formation occurred, followed by apparent mitoaggregate disassembly (*t* *=* 73–117 min). **j** Time-course quantification of mitoaggregate disassembly and LifeAct-EGFP intensity within the illuminated regions in the cell in **g**. A burst of F-actin events temporally correlated with mitoaggregate disassembly. Scale bar: 10 μm

Time-lapse imaging of mitoaggregate disassembly (dynamics of Parkin-labeled mitochondria) revealed that the process included fragmentation as well as long-range dispersion, (Supplementary Fig. 1e). This observation led us to evaluate whether cytoskeleton is involved in mitoaggregate disassembly. Interestingly, we observed F-actin formation (using the yeast-derived F-actin marker LifeAct-EGFP^11^, Fig. 2a, b and Supplementary Movie 2), as opposed to microtubule or intermediate filament generation (Supplementary Fig. 2a, b), near Parkin-labeled mitochondria. Phalloidin staining also confirmed that F-actin structures formed in proximity to the mitoaggregates, both in cells expressing and not expressing LifeAct-EGFP (Supplementary Fig. 2c-e). F-actin structures selectively appeared in the subcellular regions where Parkin-labeled mitoaggregates resided (Fig. 2c and Supplementary Fig. 3a). The same phenomenon can also be observed in HEK293 cells, a cell line that possesses high levels of endogenous Parkin (Supplementary Fig. 3b). The F-actin structures were not observed in HeLa cells lacking Parkin (Supplementary Fig. 3c), suggesting that F-actin assemblies are spatially coupled to Parkin-mediated mitophagy. The F-actin assemblies observed were transient and circular in dynamics (Fig. 2e, f), and occurred specifically during mitoaggregate disassembly (90–100 min following damage) (Fig. 2d). To better visualize their temporal overlap, we photoimpaired a small number of mitochondria within HeLa cells (Fig. 2g, h). As shown in Fig. 2i, multiple rounds of circular F-actin generation, which initially occurred ~70 min following mitochondrial damage, accompanied mitoaggregate disassembly. The total LifeAct-EGFP intensities increased concurrently with Parkin-labeled mitochondrial particle count within light-illuminated areas (Fig. 2j). All of the above support a functional link between the observed circular F-actins and mitoaggregate disassembly.

Our observed circular F-actin differ in form and function from other LifeAct-EGFP imaged F-actins during mitochondrial fission. For example, LifeAct-EGFP has been used to reveal transient actin assembly along tubular mitochondria during mitochondrial fission^12^. In our assay, the dynamics and temporal occurrence of this fission-related actin cycling, when detected, were distinct from that of circular F-actin observed during mitoaggregate disassembly (Supplementary Fig. 5a-b). These revealed that mitoaggregate disassembly-associated circular F-actins are different from those that drive mitochondrial fission.

### Actinomyosin-dependent mitoaggregate disassembly

**Fig. 3:**
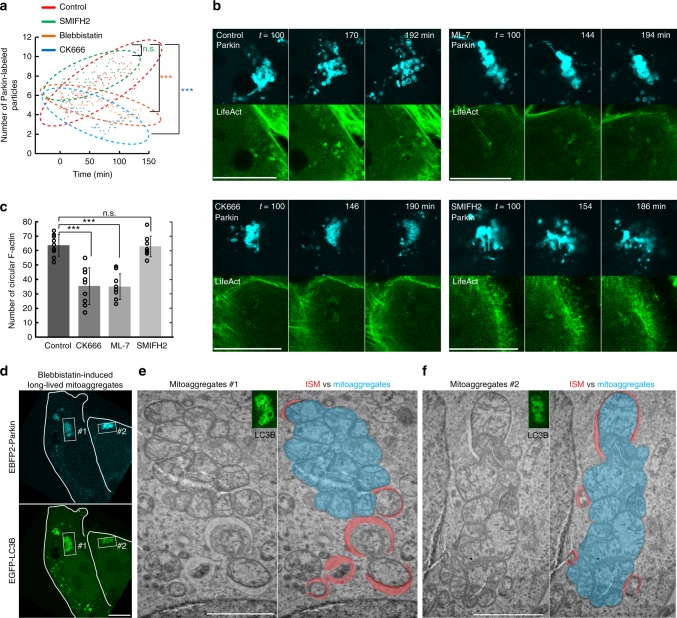
Inhibition of actinomyosin affects mitoaggregate disassembly and isolation membrane formation. **a** Quantification of mitoaggregate fragmentation levels during Parkin-mediated mitophagy under different inhibition conditions as shown in Supplementary Fig. 6a. Disassembly increases particle count over time, as shown in control cells. CK666 and blebbistatin blocked the increase, while SMIFH2 did not. *t* *=* 0 min, when Parkin signals become detectable on impaired mitochondria. (mean; *n* = 5 for control and blebbistatin, *n* = 3 for CK666 and SMIFH2 biologically independent experiments; n.s., not significant; ***p < 0.0001 as evaluated using Mann–Whitney *U* Test). **b** Representative images of mitoaggregate disassembly associated F-actin formation under different inhibition conditions as shown in Supplementary Fig. 6b. As quantified in **c**, CK666 and ML-7 impaired circular F-actin formation, but SMIFH2 did not. (mean ± S.D.; *n* = 9 biologically independent samples for all conditions; n.s., not significant; ***p < 0.0001 as evaluated using two-tailed unpaired Student’s *t*-test). **d**–**f** CLEM analysis of long-lived mitoaggregates caused by blebbistatin inhibition. **d** HeLa cells co-expressing EBFP2-Parkin, EGFP-LC3B and KR-dMito were illuminated with 559 nm to initiate mitophagy and treated with 20 μM blebbistatin 40 min after illumination. The cells were fixed 3 h after illumination and analyzed by electron microscopy. **e**, **f** Two TEM views of blebbistatin-induced long-lived mitoaggregates. The TEM images are overlaid with colored replicas to outline the observed mitoaggregates (blue) and isolation membranes (ISM, red). Insets: correlated EGFP-LC3B signals from fluorescence microscopy. Scale bar: 10 μm in **b**, **d**; 2 μm in **e**, **f**

The functional relevance of actin polymerization on mitoaggregate disassembly was further evaluated using actin nucleation factor inhibitors. Mitoaggregate disassembly (Fig. 3a and Supplementary Fig. 6a) and circular F-actin formation (Fig. 3b, c and Supplementary Fig. 6b) were both significantly inhibited by treatment with CK666^13^ (an inhibitor of the actin nucleation factor Arp2/3) but not SMIFH2^14^ (an inhibitor of the linear actin nucleation factor formin), indicating that Arp2/3-dependent actin mediates mitoaggregate disassembly. That mitoaggregate disassembly is formin-independent again confirms that the circular F-actin observed is distinct from fission-related actin (requires formin as reported)^12,15^. We also examined whether myosin II contributes to mitoaggregate disassembly, considering that interplay between myosin II and F-actin generates cellular force. Pre-treatment of cells with blebbistatin (a myosin II inhibitor)^16^ before light-induced mitophagy resulted in the formation of abnormal mitoaggregates composed of tubular mitochondria rather than grape-like clusters (Supplementary Fig. 5c-e). Given the involvement of myosin II in mitochondria fission^17^, these abnormal mitoaggregate structures likely arose from a block in mitochondria fission before mitoaggregate formation, further supporting the notion that grape-like mitoaggregates form after mitochondrial fission. To more specifically test whether myosin II partakes in mitoaggregate disassembly, we applied blebbistatin 40 min after photoillumination to allow the normal progression of mitochondrial fission that occurs immediately upon mitochondrial damage as reported^18,19^ (Supplementary Fig. 5f). Phenocopying the effect of CK666, this blebbistatin treatment led to defective disassembly of mitoaggregates (Fig. 3a). Another myosin II inhibitor, ML7, also blocked mitoaggregate disassembly and circular F-actin formation (Fig. 3b, c). All of the above point to a role of the actinomyosin system in driving mitoaggregate disassembly.

### Mitoaggregate disassembly vs. autophagy initiation

Non-disassembled mitoaggregates (through Myosin II inhibition) were labeled by EGFP-LC3B (Fig. 3d and Supplementary Fig. 7a, b) as well as by the mitophagy receptor NDP52 (Supplementary Fig. 7c-e). Meanwhile, under CLEM examination, long-lived grape-like mitoaggregates (through Myosin II inhibition) are only sparsely decorated with isolation membranes (ISMs) on their surfaces (Fig. 3e, f). Non-disassembled mitoaggregates were rarely seen to be completely sequestered into autophagosomes as demonstrated by serial CLEM sections (Supplementary Fig. 7f, g). Using mRFP-EGFP-LC3B for real-time analysis of mitophagy degradation rate (Supplementary Fig. 8a-c)^10^, non-disassembled mitoaggregates were defective in their turnover by lysosomes (Supplementary Fig. 8d-e). These results demonstrated that EGFP-LC3B/NDP52 labeling seen on myosin II-blocked long-lived mitoaggregates did not represent complete autophagic engulfment of the mitoaggregates (LC3B may have become directly conjugated onto mitochondria owing to the observed colocalization between LC3B and Parkin-labeled mitoaggregates with blebbistatin treatment. However, under these conditions mRFP-EGFP-LC3B can still be used to track the delivery of damaged mitochondria into lysosome). Also, these observations let us hypothesize that actinomyosin-dependent mitoaggregate disassembly reduces mitochondrial dimensions for efficient sequestration by autophagic membranes.

**Fig. 4:**
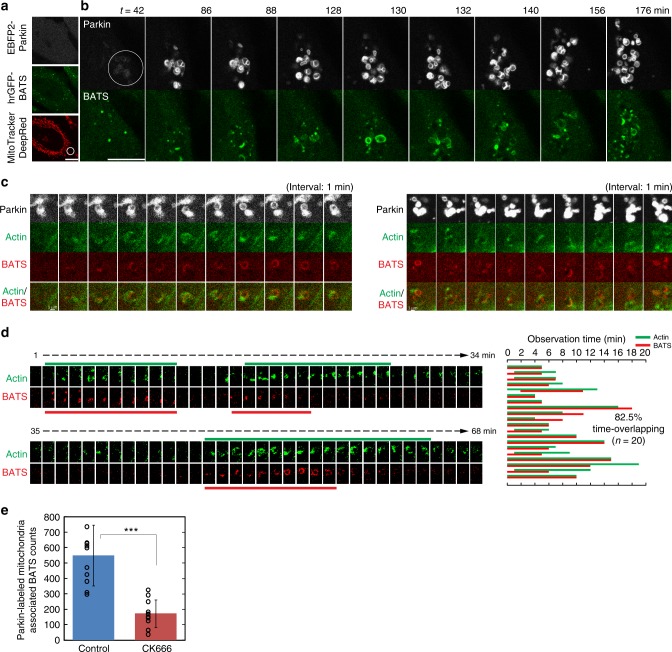
Synchronous formation between BATS and circular F-actin structures during mitoaggregate disassembly. **a** A MitoTracker DeepRed FM-stained HeLa cell co-expressing EBFP2-Parkin and hrGFP-BATS was illuminated with 635 nm (white circle) to locally activate Parkin-mediated mitophagy. **b** Selected frames from a time-lapse movie (Supplementary Movie 5) on the cellular region undergoing mitophagy in **a** after 635 nm illumination. Scale bar: 10 μm. **c** Two examples of synchronous formation of BATS and circular F-actin structures near Parkin-labeled mitoaggregates. MitoTracker DeepRed FM-stained HeLa cells co-expressing EBFP2-Parkin, LifeAct-EGFP, and TagRFP-BATS were illuminated with 635 nm to locally activate Parkin-mediated mitophagy. Scale bar: 1 μm. **d** Newly formed circular F-actin (green lines) and BATS-positive structures (red lines) are indicated on thresholded images from a time-lapse movie (Supplementary Movie 6). Circular F-actin and BATS structures formation overlapped greater than 80% in time as shown in the graph (*n* = 20 from five independent experiments). **e** Mitoaggregate-associated BATS sites are decreased under CK666 treatment. Total BATS counts near Parkin-labeled mitochondria were calculated from 200 min time-lapse images with 2 min interval (mean ± S.D.; control *n* = 11, CK666 *n* = 11 biologically independent samples; ***p < 0.0001 as evaluated using two-tailed unpaired Student’s *t*-test)

Driven by the above findings, as well as the fact that the actinomyosin system participates in multiple stages of the autophagy process^20^, we further investigated if mitoaggregate disassembly is functionally linked to autophagy initiation during Parkin-mediated mitophagy. We found that the BATS domain, the carboxyl terminus of Atg14L, displayed circular dynamics at where mitoaggregates disassembled (Fig. 4a, b). BATS marked autophagy initiation sites during mitophagy, as they colocalized with Atg14L in fixed cells on Parkin-labeled mitochondria, and were spatially associated with autophagosome formation (Supplementary Fig. 9a–c). This is consistent with the fact that BATS-labeled structures are considered autophagy initiation sites due to their colocalization with Atg16 and Beclin 1 in starved cells^21,22^. Importantly, mitophagy-associated BATS structures appeared near circular F-actin at mitoaggregates (Fig. 4c and Supplementary Fig. 9d), and displayed synchronous formation dynamics (Fig. 4d). Blocking mitoaggregate disassembly by CK666 inhibited the formation of mitoaggregate-associated BATS structures (Fig. 4e). Circular F-actin mediated mitoaggregate disassembly is therefore functionally linked to autophagosome generation against Parkin-labeled mitoaggregates.

### Retarding disassembly blocks autophagy initiation

**Fig. 5:**
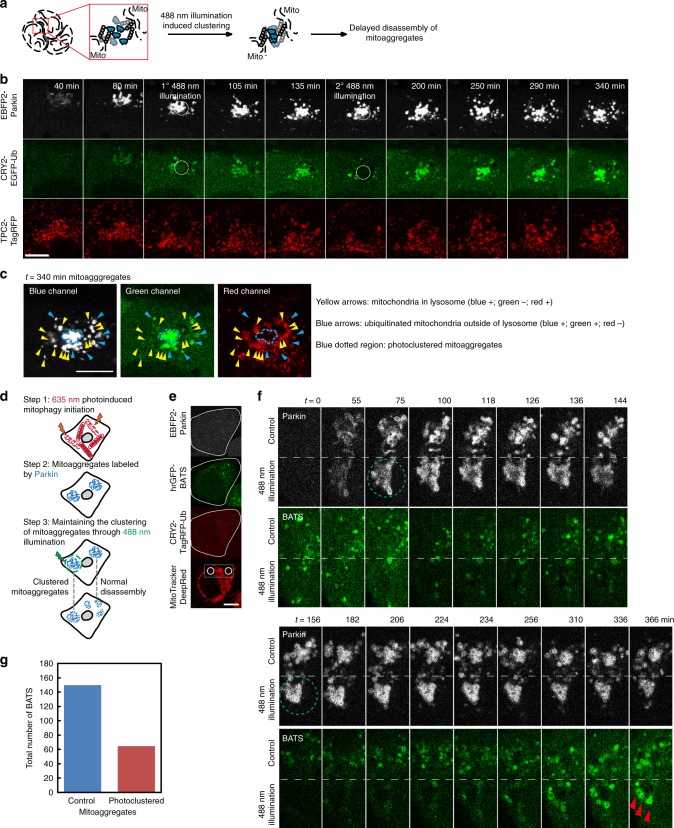
Non-disassembled mitoaggregates harbored reduced autophagosome generation and displayed delayed turnover. **a** Diagram illustrating the principle underlying photoclustering experiments. After 635 nm illumination, CRY2-Ub become conjugated to damaged mitochondria through Parkin catalyzed ubiquitination. 488 nm illumination drives CRY2 oligomerization, which helps to maintain the clustering of damaged mitochondria and delays disassembly. For clarity, representative CRY2-Ub were drawn. **b** A MitoTracker DeepRed FM-stained HeLa cell co-expressing EBFP2-Parkin, CRY2-EGFP-Ub, and TPC2-TagRFP was illuminated with 635 nm to locally activate Parkin-mediated mitophagy. Selected frames of mitoaggregates of interest 40–340 min after mitophagy initiation are shown. During mitophagy, a portion of the mitoaggregates were 488-nm illuminated (as indicated by white dotted circles) at both 80 and 140 min. **c** The progression of mitophagy in **b** 340 min after mitochondrial damage. EGFP-quenched and TPC2-positive Parkin-labeled mitochondria represents mature autophagolysosomes (yellow arrows). EGFP-fluorescence retaining and TPC2-negative Parkin-labeled mitochondria represents ubiquitinated mitochondria outside of lysosomes (blue arrows). Photoclustered mitoaggregates are outlined with a blue-dotted line. **d** Diagram illustrating the experimental flow of photoclustering experiments in single cells. **e** A MitoTracker DeepRed FM-stained HeLa cell co-expressing EBFP2-Parkin, CRY2-TagRFP-Ub, and hrGFP-BATS was illuminated with 635 nm to activate Parkin-mediated mitophagy at two separate locations (white circles) within a single cell. **f** Selected frames from a time-lapse movie (Supplementary Movie 7) on cellular region containing mitoaggregates in **e** 0–366 min after 635 nm illumination. 488 nm mediated clustering (green dotted circles) were triggered at both 77 and 154 min. The red arrows denote BATS-positive structures on the surfaces of photoclustered mitoaggregates. **g** Total number of BATS puncta in non 488-nm illuminated and 488-nm illuminated regions as shown in **f**. Scale bar: 10 μm

We further devised an optogenetic approach (Fig. 5a) to suppress disassembly through reversible crosslinking of mitoaggregate interfaces, to demonstrate that mitoaggregate disassembly is required for efficient autophagosome formation against damaged mitochondria. To achieve this, EGFP-Ub (or TagRFP-Ub) was fused to CRY2, a domain that undergoes oligomerization in response to blue light^23^. As shown in Fig. 5b, CRY2-EGFP-Ub colocalized with Parkin-labeled mitochondria 80 min after mitochondrial damage, indicating that CRY2-EGFP-Ub is able to be selectively conjugated onto mitoaggregates. The spatial enrichment of CRY2 at the mitoaggregate interfaces makes it possible to extend the duration of mitoaggregate clustering through blue light illumination. In our experiments, mitoaggregates were selectively illuminated by 488 nm light for 1 min twice, 80 and 140 min after mitochondrial damage (Fig. 5b; dotted circles). Using this scheme we found that mitoaggregates were able to remain in the clustered form for up to 5 h (Fig. 5b, c; blue dotted region). As shown in Fig. 5c, these photoclustered, long-lived mitoaggregates lacked lysosomal markers and did not display EGFP quenching (CRY2-EGFP-Ub fluorescence would be lost in acidic lysosomes). This again demonstrated that Parkin-labeled mitoaggregates cannot be efficiently targeted into lysosomes for turnover in the absence of disassembly. Next we used the optogenetic photoclustering scheme to show that a direct block on mitoaggregate disassembly affects autophagy initiation against damaged mitochondrial fragments (Fig. 5d). We damaged two separate groups of mitochondria using 635-nm light within a cell (Fig. 5e); one mitoaggregate was subsequently photoclustered using 488-nm light, while the other was left as an internal control. 488-nm illumination caused local photobleaching in the green emission channel (Fig. 5f). However, because cytosolic hrGFP-BATS freely diffuses throughout the cytoplasm, the formation of BATS-labeled autophagy initiation sites can still be monitored and counted. Unlike at the control region, which showed increased BATS-positive structures during mitoaggregate disassembly (Fig. 5f; top mitoaggregates), photoclustered mitoaggregates displayed reduced BATS counts (Fig. 5f; bottom mitoaggregates and Fig. 5g). Apparent BATS structures on photoclustered mitoaggregates only occurred 5 h following mitochondrial damage (Fig. 5f; red arrows). These BATS-sites grew only on the surfaces of photoclustered mitoaggregates, reminiscent of TEM images of blebbistatin-treated cells, where ISMs only showed up sparsely on the surface of mitoaggregates (Fig. 3e, f). Similarly, the circular F-actin counts near photoclustered mitoaggregates were reduced (Supplementary Fig. 10a, b). Collectively, these results again indicated that disassembly is not only necessary to dimensionally fit mitoaggregates into autophagic membranes but also to make them accessible to autophagy initiation.

### PIP_2_ regulates disassembly and autophagy initiation

**Fig. 6:**
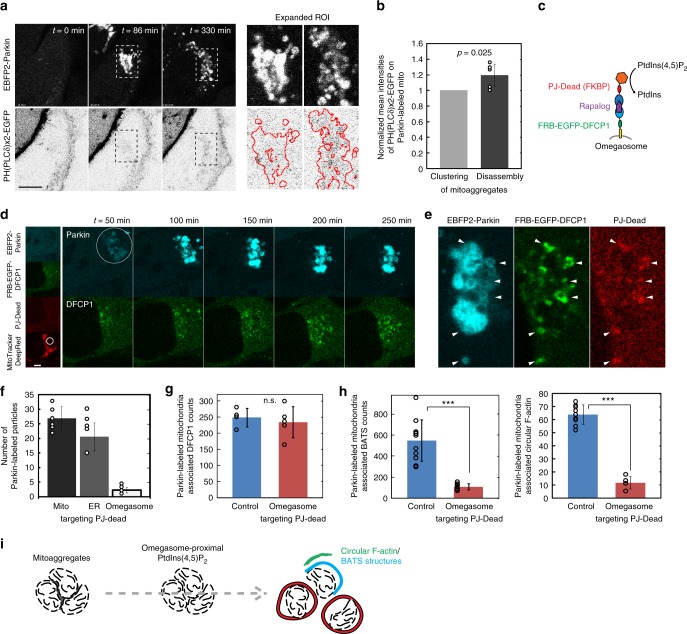
Depletion of PtdIns(4,5)P_2_ near omegasomes affects mitoaggregate disassembly. **a** A MitoTracker DeepRed FM-stained HeLa cell co-expressing EBFP2-Parkin and PH(PLCδ)x2-EGFP was illuminated with 635 nm to activate Parkin-mediated mitophagy. Increased PH(PLCδ)x2-EGFP (a PtdIns(4,5)P_2_ reporter) levels on mitoaggregates, was observed during mitoaggregate disassembly (*t* *=* 330 min) but not during the initiation (*t* = 0 min) and clustering stages (*t* = 86 min). Magnified views of the white dotted square regions are shown in right panel. **b** Intensities of PH(PLCδ)x2-EGFP on mitoaggregates during the disassembly (131–230 min after mitophagy) and clustering (30–130 min after mitophagy) phases. (mean ± S.D.; *n* = 5 biologically independent samples; *p*-value as evaluated using two-tailed unpaired Student’s *t*-test). **c** Rapalog-induced recruitment of PJ-Dead to omegasomes, leading to local depletion of PtdIns(4,5)P_2_. **d**, **e** A rapalog-treated HeLa cell co-expressing EBFP2-Parkin, PJ-Dead, and FRB-EGFP-DFCP1 was stained with MitoTracker DeepRed FM followed by 635 nm illumination (white circle) to locally initiate mitophagy. Time-lapse imaging (Supplementary Movie 8) showed that DFCP1-positive omegasomes formed near mitoaggregates. Rapalog-induced PJ-Dead recruitment to omegasomes (indicated by white arrows in **e**) resulted in the formation of long-lived, non-disassembled mitoaggregates. **f** Measurements of mitoaggregate fragmentation during Parkin-mediated mitophagy with various PJ-Dead mediated PtdIns(4,5)P_2_ depletion. (mean ± S.D.; mito *n* = 6, ER *n* = 5, omegasome *n* = 6 biologically independent samples). **g** Mitoaggregate-associated DFCP1 counts were not affected by omegasome-targeting PJ-Dead. Total DFCP1 counts near Parkin-labeled mitochondria were calculated from 250 min time-lapse images with 5 min interval (mean ± S.D.; control *n* = 4, rapalog treated *n* = 5 biologically independent samples; n.s., not significant as evaluated using two-tailed unpaired Student’s *t*-test). **h** Mitoaggregates-associated BATS/circular F-actin was decreased by omegasome-targeting PJ-Dead. Rapalog-treated HeLa cells co-expressing EBFP2-Parkin, PJ-Dead, FRB-DFCP1, and hrGFP-BATS or LifeAct-EGFP were stained with MitoTracker DeepRed FM followed by 635 nm illumination to locally initiate mitophagy. Total BATS and F-actin counts near Parkin-labeled mitochondria were calculated from 200 min time-lapse images with 2 min interval (mean ± S.D.; biologically independent samples *n* = 11 and 9 for BATS control and rapalog treated respectively; *n* = 9 and 5 for LifeAct control and rapalog treated respectively; ***p < 0.0001 as evaluated using two-tailed unpaired Student’s *t*-test). **i** Omegasome-proximal PtdIns(4,5)P_2_ regulates circular actin-based mitoaggregate disassembly and autophagy initiation. Scale bar: 10 μm

It is known that BATS domain confers binding ability to PtdIns(4,5)P_2_ in vitro^22^, and this phospholipid association is essential for Atg14 complex formation and autophagy initiation. PtdIns(4,5)P_2_ is also known to regulate F-actin formation^24^. Thus, we asked whether PtdIns(4,5)P_2_ co-regulate mitoaggregate disassembly as well as the associated BATS-site formation. Mitoaggregate-associated BATS sites were not affected by the depletion of PtdIns(3)P via treatment with 200 nM wortmannin, an inhibitor of PtdIns 3-kinases (Supplementary Fig. 11a). Meanwhile, intracellular PtdIns(4,5)P_2_ were detected near disassembled mitoaggregates through immunofluorescence (Supplementary Fig. 11b). A tandem PH domain of PLCδ fused to EGFP was also utilized to monitor intracellular PtdIns(4,5)P_2_ levels during mitophagy^25^. With the exception of the PtdIns(4,5)P_2_-enriched plasma membrane, we observed intracellular PtdIns(4,5)P_2_ generation near Parkin-labeled mitochondria during mitoaggregate disassembly in HeLa cells expressing low levels of PH(PLCδ)x2-EGFP (Fig. 6a, b), indicating a role for PtdIns(4,5)P_2_ in the regulation of mitoaggregate disassembly and BATS-site generation.

To further map the localization of mitophagic PtdIns(4,5)P_2_, we location-specifically depleted PtdIns(4,5)P_2_ using PJ-Dead, a construct containing mRFP, FKBP, and phosphatase domains^26^. Through rapalog-induced dimerization of FKBP and FRB, PJ-Dead (containing FKBP) can be selectively recruited to targeted sites expressing an FRB domain and locally catalyzed PtdIns(4,5)P_2_ to PtdIns (Supplementary Fig. 11c). Mito-targeting (FRB-EBFP-Parkin) or ER-targeting (FRB-EGFP-Sec61β) PJ-Dead did not affect mitoaggresome disassembly (Supplementary Fig. 11d,e). Remarkably, omegasome-targeting (FRB-EGFP-DFCP1) PJ-Dead impaired mitoaggregate disassembly (Fig. 6c–f). Since DFCP1-positive omegasomes are specific ER subdomains that act as platforms essential for ISM formation^9,27^ and omegasome-targeting PJ-Dead depleted PtdIns(4,5)P_2_ near mitoaggregates (Fig. 6e and Supplementary Fig. 11f, g), these results indicated that PtdIns(4,5)P_2_ proximal to omegasome regulates mitoaggregate disassembly. Omegasome-proximal PtdIns(4,5)P_2_ depletion did not affected DFCP1 recruitment to Parkin-labeled mitochondria (Fig. 6g), but impaired BATS/circular F-actin formation near mitoaggregates (Fig. 6h). These non-disassembled mitoaggregates, induced by omegasome-targeting PJ-Dead, have NDP52 recruitment (Supplementary Fig. 12a), but were defective in their delivery into lysosomes (Supplementary Fig. 12b). Altogether, our results indicated that a pool of omegasome-proximal PtdIns(4,5)P_2_ co-regulates BATS-sites/circular F-actin formation and mitoaggregate remodeling required for efficient mitophagy (Fig. 6i).

## Discussion

In this study, we discovered the presence of a pool of PtdIns(4,5)P_2_ adjacent to omegasomes, and that they coordinate mitoaggregate disassembly with autophagy initiation during Parkin-mediated mitophagy. Damaged mitochondria first become grape-like mitoaggregates. PtdIns(4,5)P_2_ then coordinates mitoaggregate disassembly with autophagy initiation to allow for efficient engulfment and turnover of impaired mitochondrial fragments.

The growing body of reports has indicated strong functional links between the actin cytoskeleton and autophagosome formation during starvation-induced autophagy^28–33^. Likewise, the mitophagy-associated F-actin could participate in the early stage of autophagosome formation owning to its synchronous formation with BATS-labeled autophagy initiation structures. Unlike CapZ-mediated PtdIns(3)P-dependent actin assembly in starvation-induced autophagy^33^, mitophagy-associated F-actin generation requires PtdIns(4,5)P_2_. We speculate that mitophagy-associated circular F-actin drive autophagosome generation by either serving as a scaffold for BATS (Atg14L) initiation structure formation or by disrupting mitoaggregates to increase the available surface accessible to ISMs growing from omegasomes. Three-dimensional structured-illumination microscopy (3D-SIM) revealed that circular F-actins represented curved sheet rather than spherical cages surrounding Parkin-labeled mitochondria (Supplementary Fig. 4a). Synchronous formation of BATS and circular F-actin near mitoaggregates indicates that it is possible to simultaneously observe circular F-actin, omegasomes, and BATS structures in TEM images. However, we failed to observe cradle-like structures^27^, which have previously been reported as omegasomal structures, and circular F-actin in our TEM images of glutaraldehyde-fixed samples. In this regard, it will be interesting to map the omegasomal structures (e.g., through the use of APEX-DFCP1) and circular F-actin in TEM images of cryogenically preserved samples to visualize transient and vulnerable structures that are not easily preserved by chemical fixation.

How may cells determine the onset of mitoaggregate disassembly? Given that Pink1-dependent phosphorylation on Parkin and Ub initiate the cascade to drive mitochondrial clustering, termination of Pink1/Parkin activity may therefore be involved in the disassembly step. Declining Pink1 activities could allow for subsequent dephosphorylation of poly-Ub and de-ubiquitination, resulting in a tendency for the mitoaggregates to disassemble. Indeed, we observed that phosphorylation levels of Ub at Ser65, a substrate of Pink1 kinase^34^, were increased on clustered mitoaggregates, and decreased upon their disassembly (Supplementary Fig. 12c, d). Therefore, it will be interesting to examine whether non-disassembled mitoaggregates [via photoclustering or depleting omegasome-proximal PtdIns(4,5)P_2_] cause delayed dephosphorylation of Ub on Ser65, or vice versa, whether delayed dephosphorylation of Ub on Ser65 (by expressing constitutively active Pink1^35,36^) affects PtdIns(4,5)P_2_-dependent mitoaggregate disassembly. FRB-DFCP1-PJ-Dead depleted PtdIns(4,5)P_2_ near omegasomes but did not disturb the PtdIns(3)P-dependent DFCP1 localization on mitophagic substrates (Fig. 6d, e, g). On the other hand, wortmannin experiments showed that depleting PtdIns(3)P did not influence PtdIns(4,5)P_2_-dependent BATS formation (Supplementary Fig. 11a). These results suggest that PtdIns(4,5)P_2_ and PtdIns(3)P signaling operate in parallel. This also suggests that PtdIns(3)P is not the sole signaling lipid for autophagy initiation; rather, PtdIns(4,5)P_2_ also plays a role, indicating another level of autophagy regulation. Increasing evidence suggests that PtdIns(4,5)P_2_ participates in autophagy, such as SNX18-dependent^37^ and Arf6-dependent^38^ autophagosome formation and Mss4-dependent mitophagy in yeast^39^. Whether PtdIns(4,5)P_2_ is locally synthesized or derived from pre-existing membrane sources remains an interesting topic for future study. Indeed, it has been reported that Atg14L interacts with PIP5KIC^40^, indicative of PtdIns conversion from PtdIns(4)P to PtdIns(4,5)P_2_ during autophagy initiation. Thus, it will also be interesting to examine whether PtdIns(4)P-enriched Golgi serves as a donor for PtdIns(4,5)P_2_ generation near omegasomes.

Why do cells go through the trouble of clustering the to-be-turned-over mitochondria, only to have them disassembled at a later stage for autophagic turnover? One reason is that this prevents the possibility of overwhelming the autophagy system, as well as reduces cellular stress. Dysfunctional mitochondria are first clustered for the reduction of cellular toxicity. Depending on the capacity and availability of the autophagy machinery, cells will then fine tune their rates of mitoaggregate disassembly so as to minimize the presence of free floating dysfunctional mitochondria in the cytoplasm. In accordance with this idea, we observed that mitoaggregate disassembly occurred at the autophagosome generating platform omegasomes, indicating that disassembly can only be as fast as the rate of autophagosome generation. In summary, our findings on the roles of omegasome-proximal PtdIns(4,5)P_2_ in Parkin-mediated mitophagy, together with recent reports of early-onset Parkinsonism-associated synaptojanin^41,42^, may underlie the pathogenic roles of PtdIns metabolism. Further efforts to reveal the interplay between PtdIns(4,5)P_2_- and PtdIns(3)P-dependent signaling axes near the omegasome will provide new insights into mitochondrial autophagy and the pathogenesis of Parkinson’s disease.

## Methods

### Plasmids, antibodies, and siRNAs

EBFP2-Parkin and TagRFP-Parkin were constructed by subcloning EBFP2 (Addgene, 14893) and TagRFP (Evrogen, FP141) into EYFP-Parkin (Addgene, 23955) respectively. KillerRed-dMito (KR-dMito) was from Evrogen (FP964). EGFP-dMito and TagRFP-dmito was constructed by PCR amplification of the mitochondrial matrix targeting sequence from KR-dMito, followed by its insertion into EGFP-C1 and TagRFP respectively. LifeAct-EGFP was generated by inserting LifeAct into pEGFP-C1. mRFP-EGFP-LC3B, EGFP-LC3B, LAMP1-YFP, hrGFP-BATS, EGFP-Atg14L, and PJ-Dead were obtained from Addgene (21074, 11546, 1816, 30496, 21635, and 38002). EB3-EGFP, EGFP-vimentin, EGFP-p62, PH(PLCδ)x2-EGFP, EGFP-DFCP1, EGFP-NDP52, and EGFP-Sec61β, and TPC2-TagRFP were constructed through PCR amplification of human genes from HeLa cell total cDNA, followed by their insertion into the pEGFP-C1 and TagRFP backbone respectively. TagRFP-BATS and TagRFP-LC3B was constructed by subcloning TagRFP into hrGFP-BATS and EGFP-LC3B. CRY2-EGFP-Ub and CRY2-TagRFP-Ub were constructed by subcloning CRY2 into EGFP-Ub (Addgene, 11928) and TagRFP-Ub. FRB-EGFP-DFCP1, FRB-EBFP2-Parkin, and FRB-EGFP-Sec61β were constructed through PCR amplification of human genes from HeLa cell total cDNA, followed by insertion into EGFP-DFCP1, EBFP2-Parkin, and EGFP-Sec61β respectively. All primer sequences used for cloning are listed in Supplementary Table 1. Alexa Fluor 647 Phalloidin was from Life Technologies (A22287). Primary antibodies used for immunofluorescence were GFP (Life Technologies, A11122, 1:500), PIP2 (Abcam, [clone 2C11] ab11039, 1:200), and phospho-Ub Ser65 (Merck, ABS1513-I, 1:500). Secondary antibodies used were Alexa Fluor 488 goat anti-mouse (Life Technologies, A11029, 1:1000) and Alexa Fluor 488 goat anti-rabbit (Life Technologies, A11034, 1:2000). Small interfering RNAs used were p62 siRNA (Thermo Scientific, M-010230-00-0005).

### Cell culture and transfection

HeLa cells (ATCC, CCL-2) and HEK293 (ATCC, CRL-1573) were cultured in DMEM medium (Life Technologies, 11965) supplemented with 10% FBS (Life Technologies, 10437) and 1% penicillin/streptomycin (Life Technologies, 15140), and maintained at 37 ^°^C and 5% CO_2_. DAPI staining was used to ensure the absence of mycobacterium contamination. Cells were transfected using Lipofectamine 2000 (Life Technologies, 11668) according to manufacturer’s instructions. For knockdown experiments, siRNAs were transfected twice into HeLa cells with 24 h intervals through Lipofectamine RNAiMAX (Life Technologies, 13778075) or Lipofactamine 2000 based on manufacturer’s protocol. Cell staining with MitoTracker Deep Red FM (Life Technologies, M22426): 100 nM in complete medium for 30 min at 37 ^°^C. Blebbistatin (Enzo Life Sciences, BML-El315) was used at 20 μM (added into the cell medium 40–50 min post induction of mitophagy or before induction of mitophagy). ML-7 (Enzo, BML-EI197) was used at 30 μM. CK666 (Enzo Life Sciences, ALX-270-506) was used 200 μM (applied 1 h before light-assisted mitophagy). SMIFH2 (Merck millipore, 344092) was used at 25 μM (applied before light-assisted mitophagy). Wortmannin (Sigma, W1628) was used 200 nM before light-assisted mitophagy. Rapalog (Clontech #635055) was used at 500 nM before light-assisted mitophagy.

### Live cell manipulation and time-lapse microscopy

Light-assisted Parkin-mediated mitophagy was performed and imaged on an Olympus FV1000 confocal microscope (60 × , numerical aperture = 1.2 water objective) equipped with a SIM scanner. Live HeLa cells were maintained under 37 ^°^C and 5% CO_2_ on the microscope for manipulation and observation (Tokai Hit, #MIU-IBC). To achieve light-assisted Parkin-mediated mitophagy, we point-scanned either 30 μW 559 nm (for KR-dMito) or 100 μW 635 nm (for MitoTracker DeepRed FM) light in chosen regions of interests in HeLa cells using Olympus FV1000’s tornado scanning. To photo-maintain mito-aggregation, we point-scanned 488 nm light over chosen mitoaggregates (labeled by EBFP2-Parkin) of interest in HeLa cells using Olympus FV1000’s tornado scanning. A zero drift compensator module (Olympus) was used to correct sample drift in the z direction during live cell imaging. Images were adjusted for brightness and compiled into time-lapse images using Image J (National Institutes of Health).

### Immunofluorescence

Cells cultured on ibidi gridded glass bottom dishes (81168) were fixed with 4% paraformaldehyde (EMS, 15711) at room temperature for 10 min, permeabilized with 0.2% (v/v) Triton X-100 in PBS (pH 7.4) containing 2% BSA at 4 ^°^C for 10 min, blocked with 0.02% (v/v) Triton X-100 in PBS containing 2% BSA at room temperature for 30 min, and incubated with primary antibodies following manufacturers’ recommendations (phalloidin AF647). For EGFP-Atg14 immunostaining, cell were fixed with 4% paraformaldehyde at room temperature for 10 min, permeabilized and blocked with 0.2% (v/v) Triton X-100 in PBS (pH 7.4) containing 2% BSA at 4 ^°^C for 1 h, immunostained with primary antibodies in PBS buffer containing 0.02% (v/v) Triton X-100 and 2% BSA overnight at 4 ^°^C, and probed with secondary antibodies (1:2000) in PBS buffer containing 0.02% (v/v) Triton X-100 and 2% BSA at room temperature for 1 h. For PIP2 immunostaining, cells cultured on ibidi gridded glass bottom dishes were fixed with 4% paraformaldehyde and 0.2% glutaraldehyde (Sigma-Aldrich, G5882) at room temperature for 15 min, permeabilized and blocked with 0.5% (v/v) Saponin in PBS (pH 7.4) containing 2% BSA at 4 ^°^C for 45 min, immunostained with primary antibodies in PBS buffer containing 0.1% (v/v) Saponin and 2% BSA overnight at 4 ^°^C, probed with secondary antibodies (1:1000) in PBS buffer containing 0.1% (v/v) Saponin and 2% BSA at 4 ^°^C for 1 h, and post-fixed with 2% paraformaldehyde at 4 ^°^C for 10 min. For phospho-Ub Ser65 immunostaining, cells cultured on ibidi gridded glass bottom dishes were fixed with 4% paraformaldehyde at room temperature for 10 min, permeabilized and blocked with 0.2% (v/v) Triton X-100 in PBS (pH 7.4) containing 2% BSA at 4 ^°^C for 1 h, immunostained with primary antibodies in PBS buffer containing 0.02% (v/v) Triton X-100 and 2% BSA at 4 ^°^C for 1 h, and probed with secondary antibodies (1:2000) in PBS buffer containing 0.02% (v/v) Triton X-100 and 2% BSA at room temperature for 1 h. Images were captured on an Olympus FV1000 confocal microscope.

### Fragmentation analysis and BATS/actin, DFCP1-sites, or PH(PLCδ)x2-EGFP intensity calculation

To count the number of EBFP2-Parkin labeled mitochondrial particles, images were thresholded, followed by particle identification using the Analyze Particles function in Image J. To count the number of BATS or DFCP1 structures near mitophagic substrates, regions containing EBFP2-Parkin labeled mitochondria were defined through the Color Threshold function in Image J. The total counts of BATS or DFCP1 within these defined regions were measured using the Analyze Particles function in Image J. The total counts of BATS were binned into 10 min intervals to calculate the total number of BATS as shown in Fig. 5g. The total number of circular actin was manually calculated. The raw intensities of PH(PLCδ)x2-EGFP within EBFP2-Parkin labeled mitochondria were calculated through the Measure function in Image J and were divided by area of EBFP2-Parkin labeled mitochondria to calculate mean PH(PLCδ)x2-EGFP intensities on mitoaggregates.

### Quantifying mitophagosome maturation through mRFP-EGFP-LC3B

Quantification of mRFP-EGFP-LC3B fluorescence on EBFP2-Parkin labeled mitochondria was performed using Image J. In brief, regions containing EBFP2-Parkin labeled mitochondria were defined through the Color Threshold function in Image J. The total mRFP and EGFP signals within these identified regions were measured using the Measure function in Image J.

### Correlative light and electron microscopy

HeLa cells seeded on MatTek gridded glass (P35G-1.5-14-CGRD-D) bottom dishes co-expressing EBFP2-Parkin, EGFP-LC3B, and KR-dMito were 559 nm illuminated to activate for mitophagy. Cells were first fixed with 4% paraformaldehyde at room temperature for 30 min and imaged under a confocal microscope (Olympus FV1000). The samples were then fixed in 2.5% glutaraldehyde in 0.1 M sodium cacodylate buffer (EMS, 11652, pH 7.4) at 4 ^°^C for 16 h, post-fixed in 1% osmium tetroxide (EMS, 19150) at room temperature for 1 h, dehydrated, and embedded in Low Viscosity Embedding Kit (EMS, #14300). They were further trimmed and sliced to 70 nm thickness sections using ultramicrotome (Leica Ultracut UCT). Sections were stained with uranyl acetate (EMS, 22400) and lead citrate (EMS, #17810) before imaging under TEM (Hitachi H7000).

### Structured-illumination microscopy

HeLa cells seeded on ibidi Grid-500 glass bottom (81168) dishes co-expressing EBFP2-Parkin, LifeAct-EGFP, and KR-dMito were 559 nm illuminated to activate for mitophagy. Cells were time-lapse imaged under a confocal microscope, and fixed with 4% paraformaldehyde when circular F-actin assemblies appear around Parkin-labeled mitochondria. The fixed samples were further imaged under a Zeiss ELYRA S1.

### Statistical analysis

No statistical methods were used to predetermine sample size. Experiments were not randomized, and the investigators were not blinded to allocation during experiments and outcome assessment.

## Supplementary Information

Supplementary Informartion

Supplementary Movie 1

Supplementary Movie 2

Supplementary Movie 3

Supplementary Movie 4

Supplementary Movie 5

Supplementary Movie 6

Supplementary Movie 7

Supplementary Movie 8

## Data Availability

The data sets generated from this manuscript are available from the corresponding author upon reasonable request.
