# Supplementary material for: Omegasome-proximal PtdIns(4,5)P2 couples F-actin mediated mitoaggregate disassembly with autophagosome formation during mitophagy
Source: Nat Commun. 2019 Feb 27;10:969. doi: 10.1038/s41467-019-08924-5 (PMC6393429; doi:10.1038/s41467-019-08924-5)
Supplement: Supplementary file 1 — Supplementary Informartion [file 41467_2019_8924_MOESM1_ESM.pdf]

Supplementary Information

Omegasome-proximal PtdIns(4,5)P_2 couples F-actin mediated mitoaggregate disassembly with autophagosome formation during mitophagy

Hsieh et al.

Supplementary Figure 1 (related to Fig. 1).
Supplementary Figure 2 (related to Fig. 2).
Supplementary Figure 3 (related to Fig. 2).
Supplementary Figure 4 (related to Fig. 2).
Supplementary Figure 5 (related to Fig. 3).
Supplementary Figure 6 (related to Fig. 3).
Supplementary Figure 7 (related to Fig. 3).
Supplementary Figure 8 (related to Fig. 3).
Supplementary Figure 9 (related to Fig. 4).
Supplementary Figure 10 (related to Fig. 5).
Supplementary Figure 11 (related to Fig. 6).
Supplementary Figure 12 (related to Fig. 6).
Supplementary Table 1. Primers

Hsieh et al. Supplementary information. Supplementary Figure 1

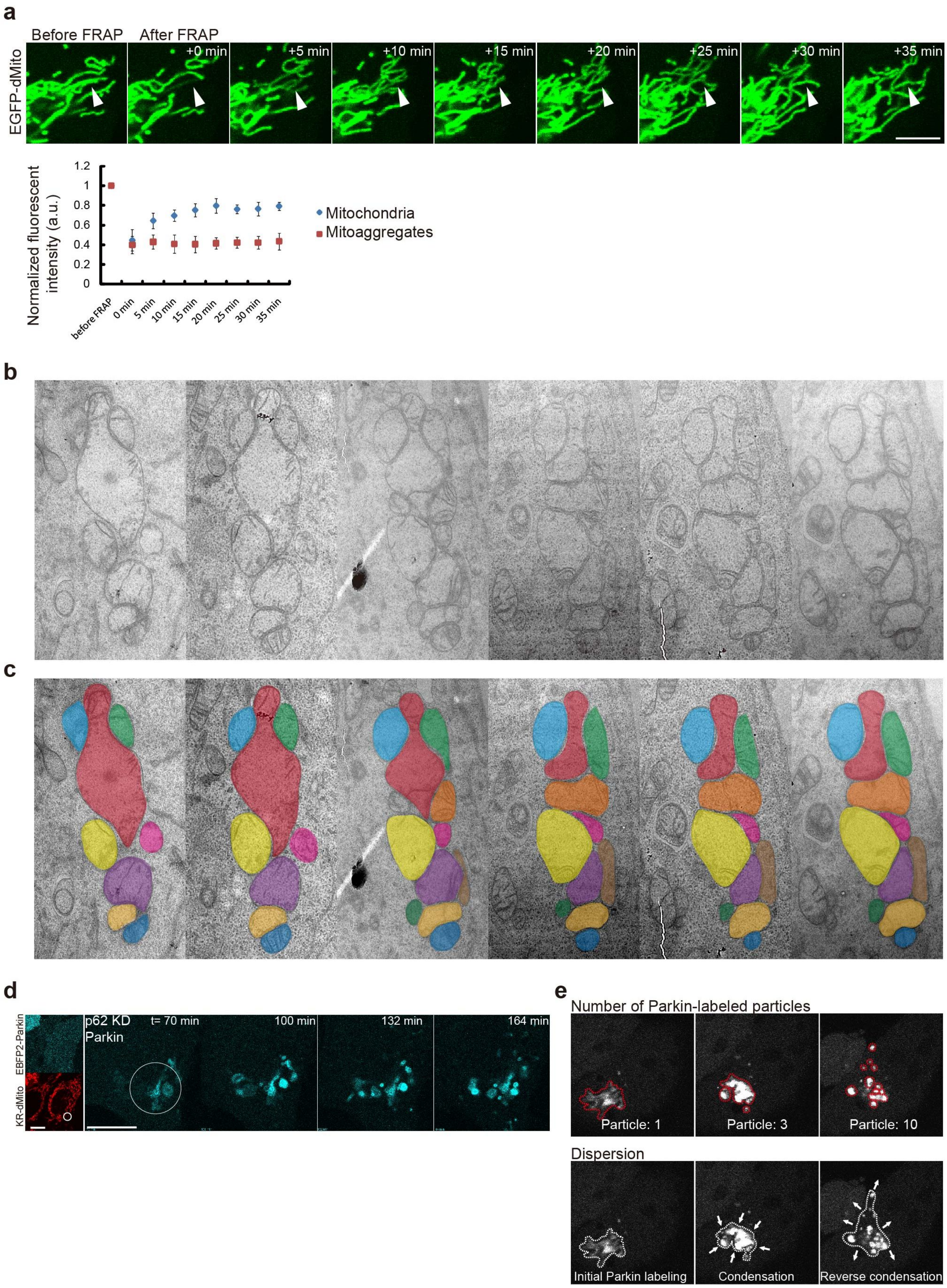

Supplementary Figure 1. Formation and disassembly of p62-dependent grape-like mitoaggregates. (**a**) Analysis of mitochondrial connectivity by FRAP. HeLa cells expressing EGFP-dMito were photobleached by 488-nm light within a small portion of mitochondria (indicated by white arrow) and monitored for recovery over a course of 35 min. *Graph*: Fluorescence recovery of EGFP-dMito from Parkin-labeled mitoaggregates and healthy mitochondria. (mean±S.D.; mitoaggregates *n*=5, healthy mitochondria *n*=5 biologically independent samples). Scale bar: 5 μm. (**b**) TEM images on serial sections of mitoaggregates 90 min after mitochondrial damage. (**c**) Colored replica of mitoaggregates in **b** outlining individual mitochondrial fragments. (**d**) In the absence of p62, Parkin-labeled mitochondria did not cluster. HeLa cells transfected with EBFP2-Parkin, KR-dMito, and p62 siRNA were 559-nm illuminated (white circle) for mitophagy initiation. *Right panel*: Magnified view of the cell 70-164 min after mitophagy initiation. Scale bar: 10 μm. (**e**) Mitoaggregate fragmentation and dispersion during disassembly.

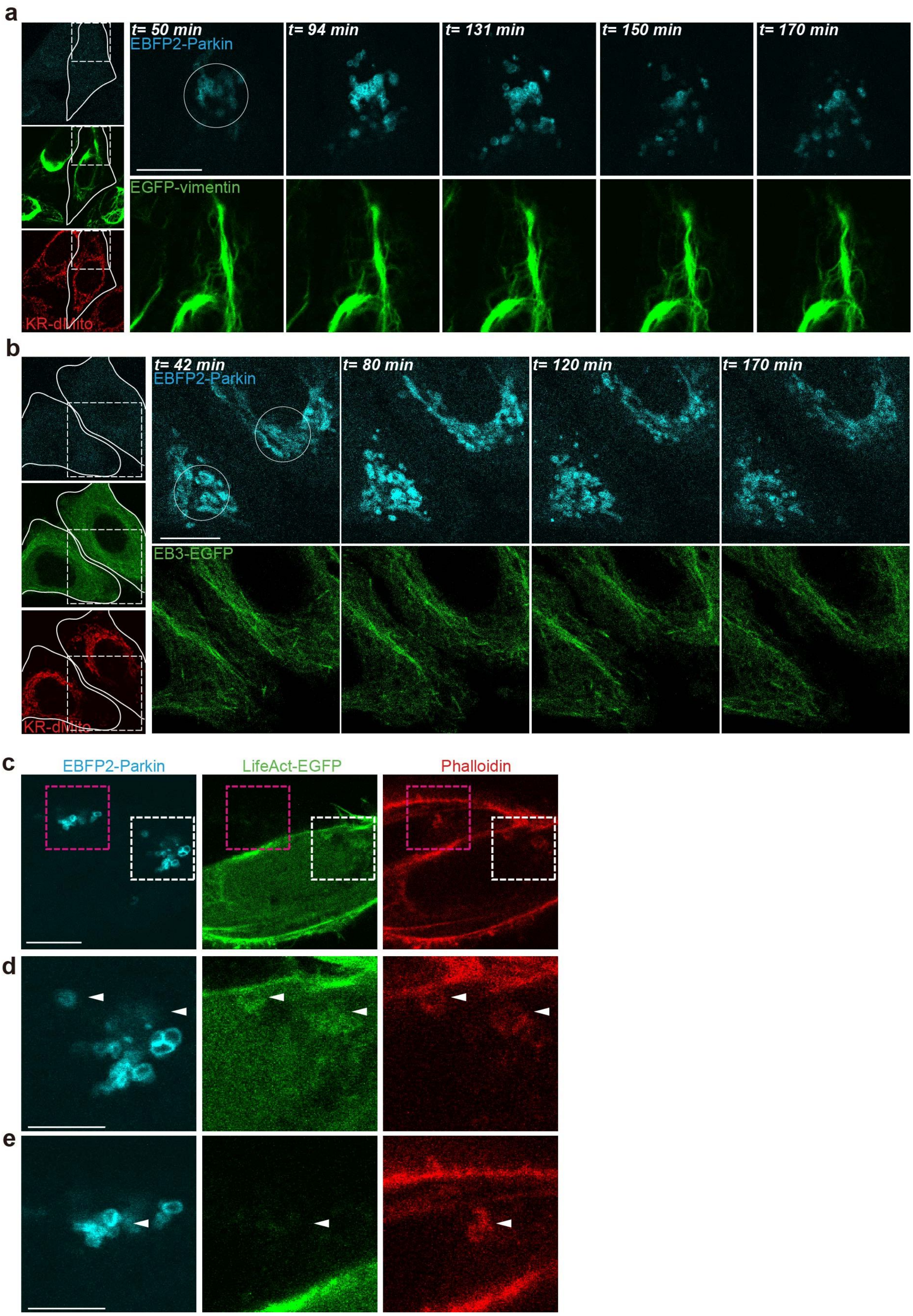

Supplementary Figure 2. Circular F-actin, but not microtubules and intermediate filaments, formed at sites of mitoaggregate disassembly. HeLa cells co-expressing EBFP2-Parkin, KR-dMito, and EGFP-vimentin (in **a**) or EB3-EGFP (in **b**) were illuminated with 559 nm (white circle) to initiate mitophagy. *Right panels*: Magnified view of the white-dotted square region within 170 min after the induction of Parkin-mediated mitophagy. Both vimentin and EB3 did not display apparent changes after local mitophagy initiation. (**c**) HeLa cells co-expressing EBFP2-Parkin, KR-dMito, and LifeAct-EGFP were illuminated with 559 nm to initiate mitophagy. The cells were fixed and stained with AlexaFluor 647-Phalloidin after Parkin-labeled mitochondria became detectable in both cells. Scale bar: 10 μm (**d-e**) Magnified views of the white (in **d**) and pink (in **e**) dotted square regions. LifeAct-EGFP was not detectable in the *top-left* cell, while the *bottom-right* cell displayed strong expression. Both cells displayed highly curved phalloidin-positive F-actin structures near Parkin-labeled mitochondria (white arrows; this indicated that these structures were not artificially induced through LifeAct-EGFP overexpression). LifeAct-GFP signals overlapped with phalloidin staining (*bottom-right* cell). Scale bar: 5 μm

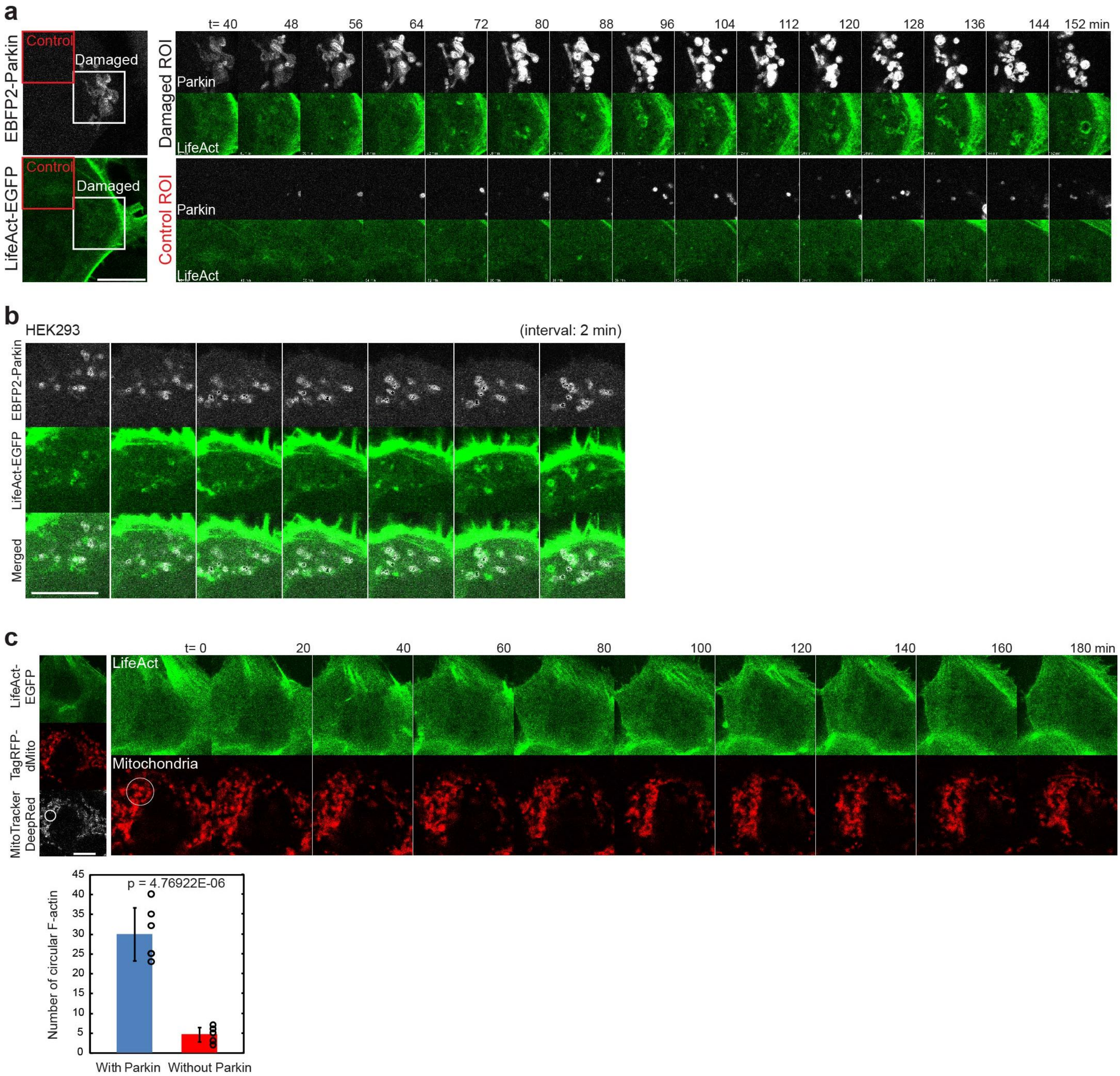

Supplementary Figure 3. Parkin-mediated mitophagy associated circular F-actin formation. (**a**) Time-lapse analysis on the spatial preference for circular F-actin formation during mitophagy. Magnified views of the illuminated region (white square) and non-illuminated region (red square) 40-152 min after mitophagy initiation are shown in the right panels. Scale bar: 10 μm. (**b**) Mitophagy-associated circular F-actin formation in HEK293 cell. MitoTracker DeepRed FM-stained HEK293 cells co-expressing EBFP2-Parkin and LifeAct-EGFP were illuminated with 635 nm to locally activate Parkin-mediated mitophagy. Selected frames were showing the cellular region undergoing mitophagy and circular F-actin formation. Scale bar: 10 μm. (**c**) Time-lapse analysis of circular F-actin formation in the absence of Parkin. MitoTracker DeepRed FM-stained HeLa cells co-expressing TagRFP-dMito and LifeAct-EGFP were illuminated with 635 nm (white circle; immediate loss of MitoTracker DeepRed FM fluorescence) to generate mitochondrial damage. Selected frames after mitochondrial damage are shown in the right panels. *Graph*: Total number of circular F-actin structures that formed within 200 min after photoinduced-mitochondrial damage in the presence or absence of Parkin. (mean±S.D.; with Parkin *n*=6, without Parkin *n*=6 biologically independent samples; p-value as evaluated using two-tailed unpaired Student's *t*-test). Scale bar: 10 μm.

a

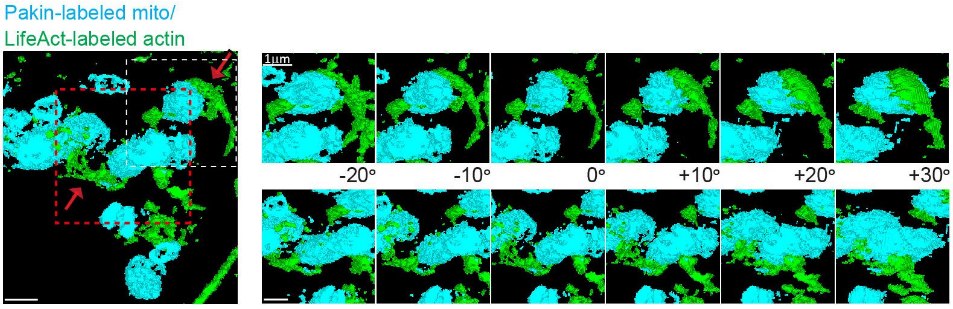

Supplementary Figure 4. Ultrastructures of circular F-actin during mitoaggregate disassembly. (**a**) 3D views of circular F-actin obtained by 3D-SIM. F-actin (LifeAct-EGFP; green) appeared as highly curved structures that formed near Parkin-labeled mitochondria (EBFP2-Parkin; cyan). Scale bar: 1 μm

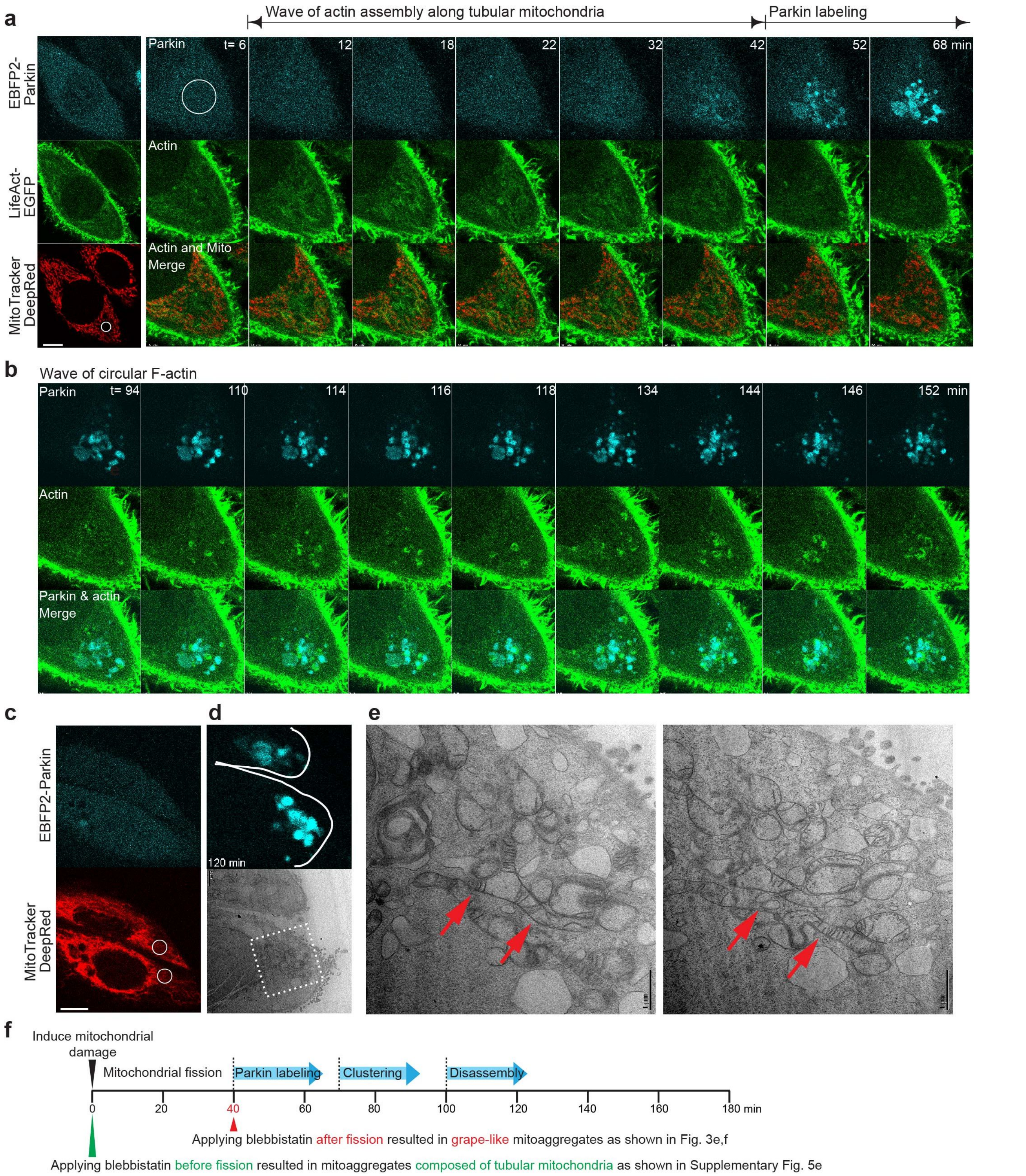

Supplementary Figure 5. Mitochondrial fission-associated actin cycling appears prior to the formation of circular F-actin. (**a-b**) Two forms of actin assembly were observed at different time points with distinct dynamics following mitochondrial damage. (**a**) A MitoTracker DeepRed FM-stained HeLa cell co-expressing EBFP2-Parkin, LifeAct-EGFP was illuminated with 635 nm to locally activate Parkin-mediated mitophagy (white circle). *Right panels*: Selected frames from a time-lapse movie (Supplementary Movie 4) on the cellular region undergoing mitophagy 6-68 min after 635 nm illumination. Before detectable Parkin-labeling (*t=42 min*), a cycling actin wave (*t=12-32 min*) along tubular mitochondria (red, MitoTracker DeepRed) was observed. (**b**) Selected frames from a time-lapse movie (Supplementary Movie 4) on the cell in **a** 94-152 min after 635 nm illumination. Circular F-actin, but not cycling actin waves, were observed during mitoaggregate disassembly. Scale bar: 10 μm. (**c-f**) Inhibiting mitochondrial fission before mitochondrial damage led to the formation of mitoaggregates comprised of entangled tubular mitochondria (red arrows) rather than grape-like structures. (**c**) MitoTracker DeepRed FM-stained HeLa cells expressing EBFP2-Parkin were pre-treated with blebbistatin, then were illuminated with 635 nm (white circles) to locally activate Parkin-mediated mitophagy. The cells were fixed 120 min after illumination and analyzed by fluorescence microscopy (**d**, *top*) and electron microscopy (**d**, *bottom*). Scale bar: 10 μm. (**e**) Magnified TEM views of the white dotted square region in **d**. Abnormal mitoaggregates composed of tubular mitochondria were observed (red arrows). Scale bar: 1 μm. (**f**) Diagram illustrating changes in mitochondrial morphology at the indicated time points. Applying blebbistatin before or after mitochondrial fission caused the formation of different mitoaggregate structures.

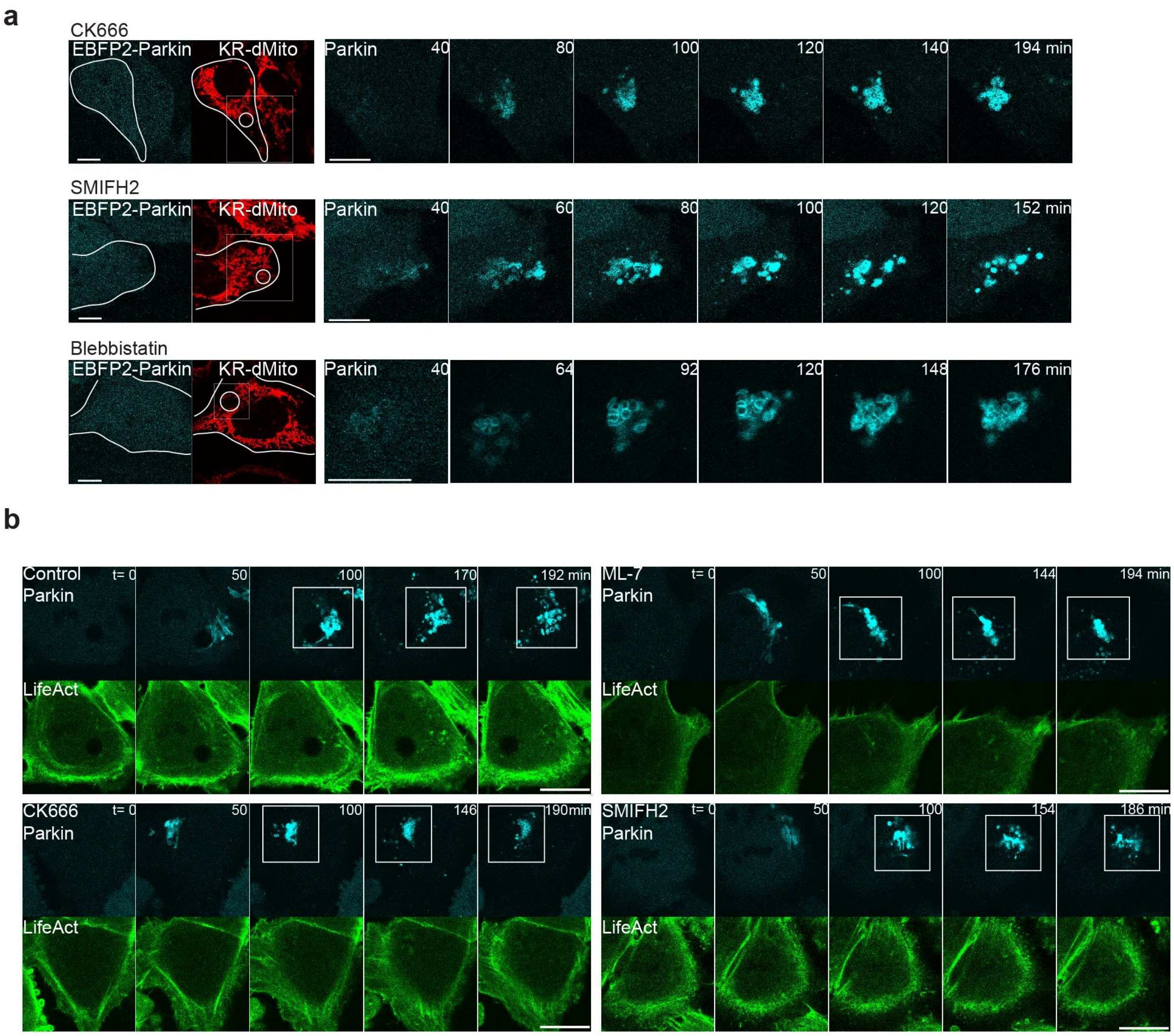

Supplementary Figure 6. Actinomyosin is required for mitoaggregate disassembly and circular F-actin assembly. (**a**) Inhibition of Arp2/3-driven actin polymerization by CK666 or the inhibition of myosin II by blebbistatin blocked disassembly of mitoaggregates in HeLa cells co-expressing EBFP2-Parkin and KR-dMito. Inhibition of formin-based linear actin polymerization by SMIFH2 did not. *Right panels*: Selected frames of the white square region. (**b**) Inhibition of Arp2/3 by CK666 or inhibition of myosin II by ML-7 affects circular F-actin formation during mitoaggregate disassembly. MitoTracker DeepRed FM-stained HeLa cells co-expressing EBFP2-Parkin and LifeAct-EGFP were illuminated with 635 nm to locally activate Parkin-mediated mitophagy while treated with indicated inhibitors. Selected frames are shown in right panels and magnified views of white square regions are shown in Fig. 3b. Scale bar: 10 μm

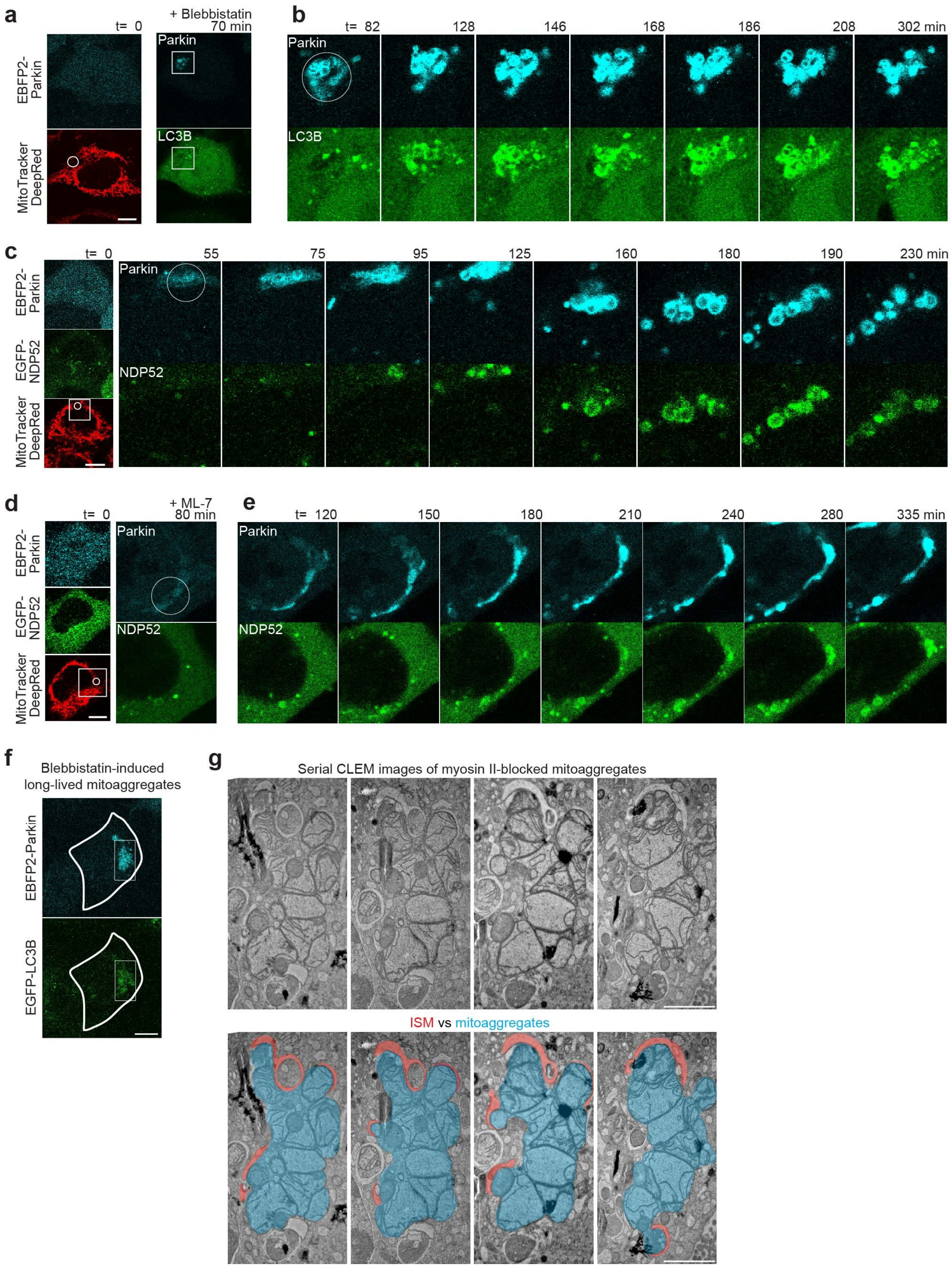

Supplementary Figure 7. Myosin II inhibition blocks mitoaggregate disassembly and ISM formation. (**a-b**) A MitoTracker DeepRed FM-stained HeLa cell co-expressing EBFP2-Parkin and EGFP-LC3B was illuminated with 635 nm (white circle) to initiate mitophagy. The cell was then treated with 20 μM blebbistatin 40 min after illumination, leading to impaired mitoaggregate disassembly and pan-LC3B labeling as shown in **b**. Scale bar: 10 μm. (**c-e)** NDP52 labeling on mitoaggregates under normal and myosin II-blocked conditions. MitoTracker DeepRed FM-stained HeLa cells co-expressing EBFP2-Parkin and EGFP-NDP52 were illuminated with 635 nm (white circle) to initiate mitophagy. Similar to the control (in **c**), ML7-blocked mitoaggregates (in **d-e**) displayed NDP52 labeling. Scale bar: 10 μm. (**f-g**) CLEM analysis of long-lived mitoaggregates caused by myosin II inhibition. (**f**) The HeLa cell co-expressing EBFP2-Parkin, EGFP-LC3B, and KR-dMito was 559-nm illuminated for mitophagy initiation and treated with 20 μM blebbistatin 40 min after illumination. The cell was fixed 4 hr after illumination and analyzed by electron microscopy. Scale bar: 10 μm. (**g**) TEM views on serial sections of blebbistatin-induced long-lived mitoaggregates (white square in **f**). The TEM image is overlaid with a colored replica to outline the observed mitoaggregate (blue) and isolation membranes (ISM, red). ISMs grew only on the surfaces of the mitoaggregate (4 hr after mitophagy initiation). Scale bar: 2 μm

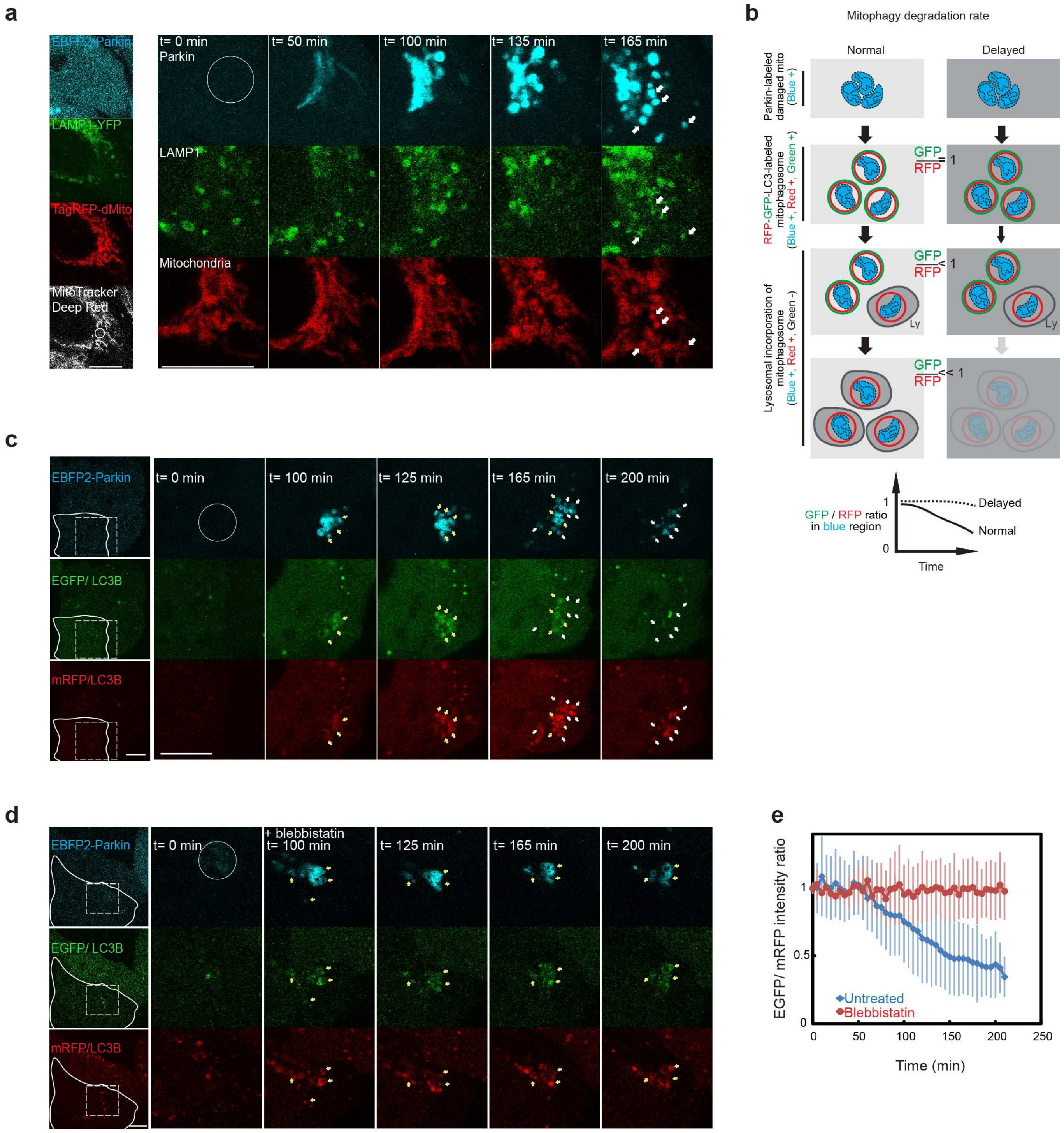

Supplementary Figure 8. Blocking mitoaggregate disassembly with blebbistatin resulted in delayed turnover of damaged mitochondria. (**a**) A MitoTracker DeepRed FM-stained HeLa cell co-expressing EBFP2-Parkin, LAMP1-YFP, and TagRFP-dMito was illuminated with 635 nm (white circle) to initiate mitophagy. Right panels show selected frames of Parkin-mediated mitophagy and demonstrate that Parkin-labeled damaged mitochondria were delivered into lysosomes (indicated by white arrows). (**b**) Monitoring clearance of damaged mitochondria using mRFP-GFP-LC3B. (**c-e**) Turnover of Parkin-labeled mitochondria. HeLa cells co-expressing EBFP2-Parkin and mRFP-EGFP-LC3B and stained with MitoTracker DeepRed FM were illuminated with 635 nm in the white circles, then left untreated (in **c**) or treated with blebbistatin (in **d**) 40 min post-light activation. *Right panels*: magnified view of the white-dotted square regions 0-200 min after mitochondrial damage. In the untreated cell, autophagosomes assembled around Parkin-labeled mitochondria (yellow arrows) and were able to mature (quenching of EGFP signals on LC3B; white arrows). (**e**) Quantifying mRFP-EGFP-LC3B. EGFP/mRFP signal ratios on Parkin-labeled mitochondria under different treatment conditions (*t=0 min*: when Parkin signals became detectable on impaired mitochondria) in HeLa cells co-expressing EBFP2-Parkin and mRFP-EGFP-LC3B (as in **c** and **d**). Loss of EGFP signals indicates delivery of impaired mitochondria into autolysosomes. Blebbistatin treatment led to delayed delivery (red curve) as compared to untreated controls (blue curve). (mean±S.D.; untreated *n*=5, blebbistatin treated *n*=5 biologically independent samples). Scale bar: 10 μm.

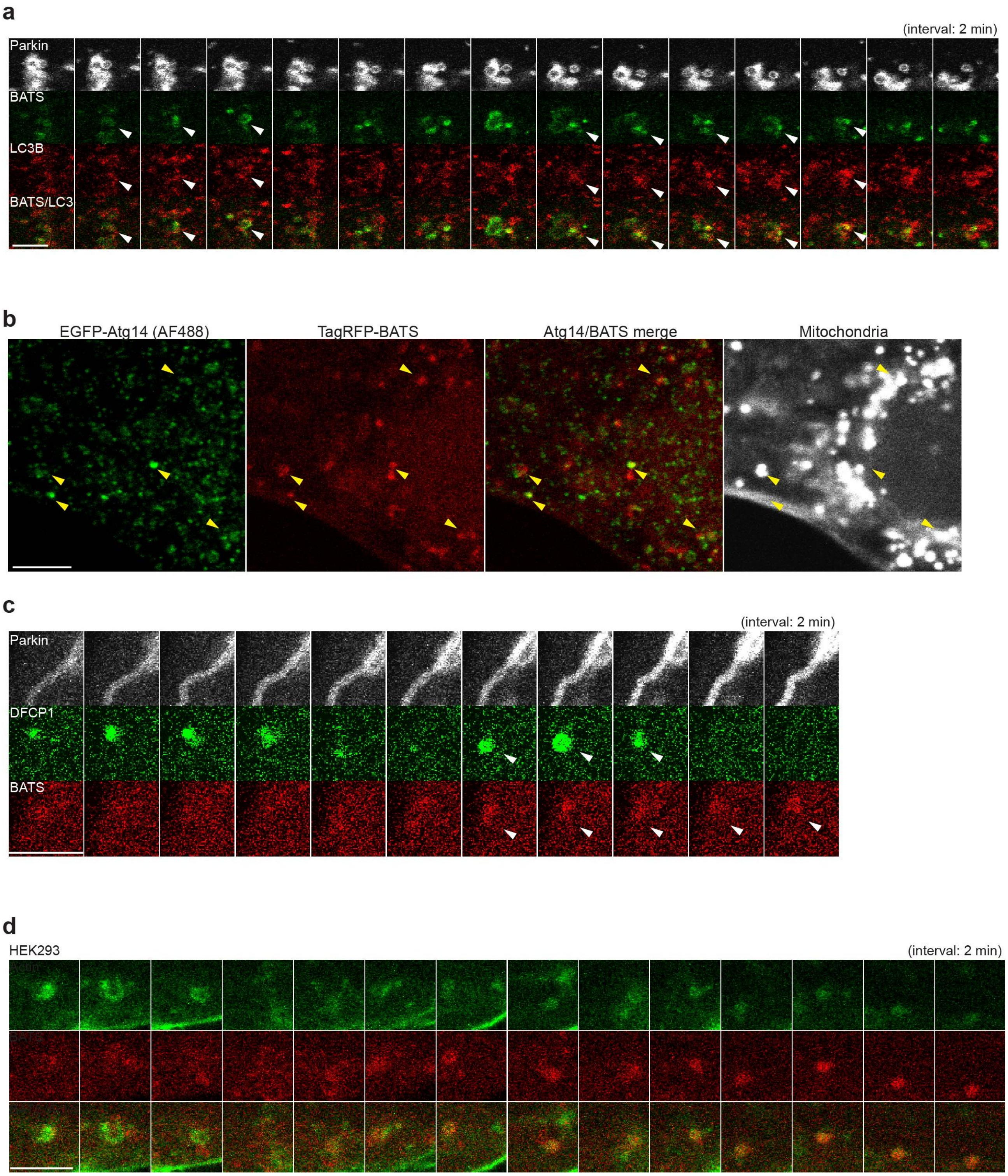

Supplementary Figure 9. BATS-labeled structures represent autophagy initiation sites. (**a**) A MitoTracker DeepRed FM-stained HeLa cell co-expressing EBFP2-Parkin, hrGFP-BATS, and TagRFP-LC3B was illuminated with 635 nm to initiate mitophagy. Selected frames of Parkin-mediated mitophagy demonstrate autophagosome formation from BATS-labeled sites (indicated by white arrows). (**b-c**) BATS-labeled sites represent autophagy initiation sites. MitoTracker DeepRed FM-stained HeLa cells co-expressing EBFP2-Parkin, TagRFP-BATS and EGFP-Atg14 (in **b**) or EGFP-DFCP1 (in **c**) were illuminated with 635 nm to initiate mitophagy. EGFP-Atg14 foci were probed using primary antibody against GFP. BATS-labeled sites colocalized with Atg14 (in **b**, yellow arrows) and DFCP1 (in **c**, white arrows) on Parkin-labeled mitochondria. (**d**) Synchronous formation of BATS and circular F-actin structures in HEK293 cells. MitoTracker DeepRed FM-stained HEK293 cells co-expressing LifeAct-EGFP and TagRFP-BATS were illuminated with 635 nm to locally activate Parkin-mediated mitophagy. Scale bar: 5 μm

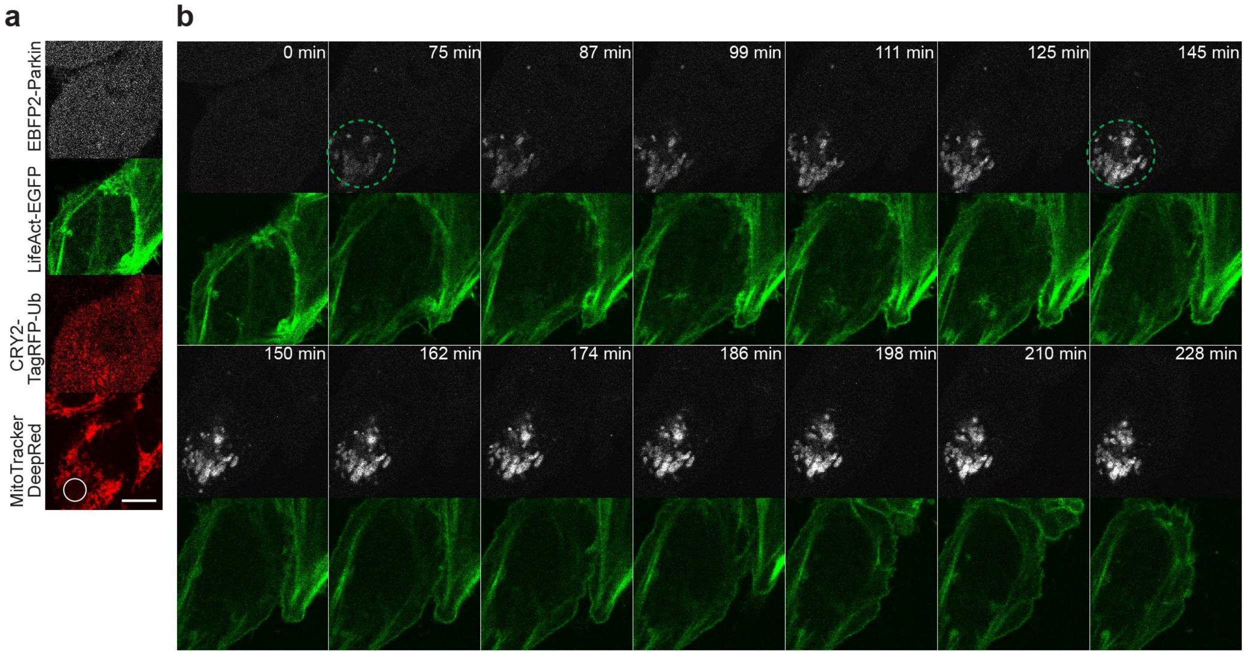

Supplementary Figure 10. Photoclustered mitoaggregates do not disassemble and triggered less circular F-actin formation. (**a**) A MitoTracker DeepRed FM-stained HeLa cell co-expressing EBFP2-Parkin, CRY2-TagRFP-Ub, and LifeAct-EGFP was illuminated with 635 nm to activate Parkin-mediated mitophagy (white circle). (**b**) Selected frames on the cellular region containing damaged mitochondria in **a** 0-228 min after 635 nm illumination. 1st and 2nd photoclustering (indicated by green dotted circles) were carried out at 75 and 145 min after 635 nm illumination, respectively. No apparent circular F-actin formation was observed near photoclustered mitoaggregates. Scale bar: 10 μm

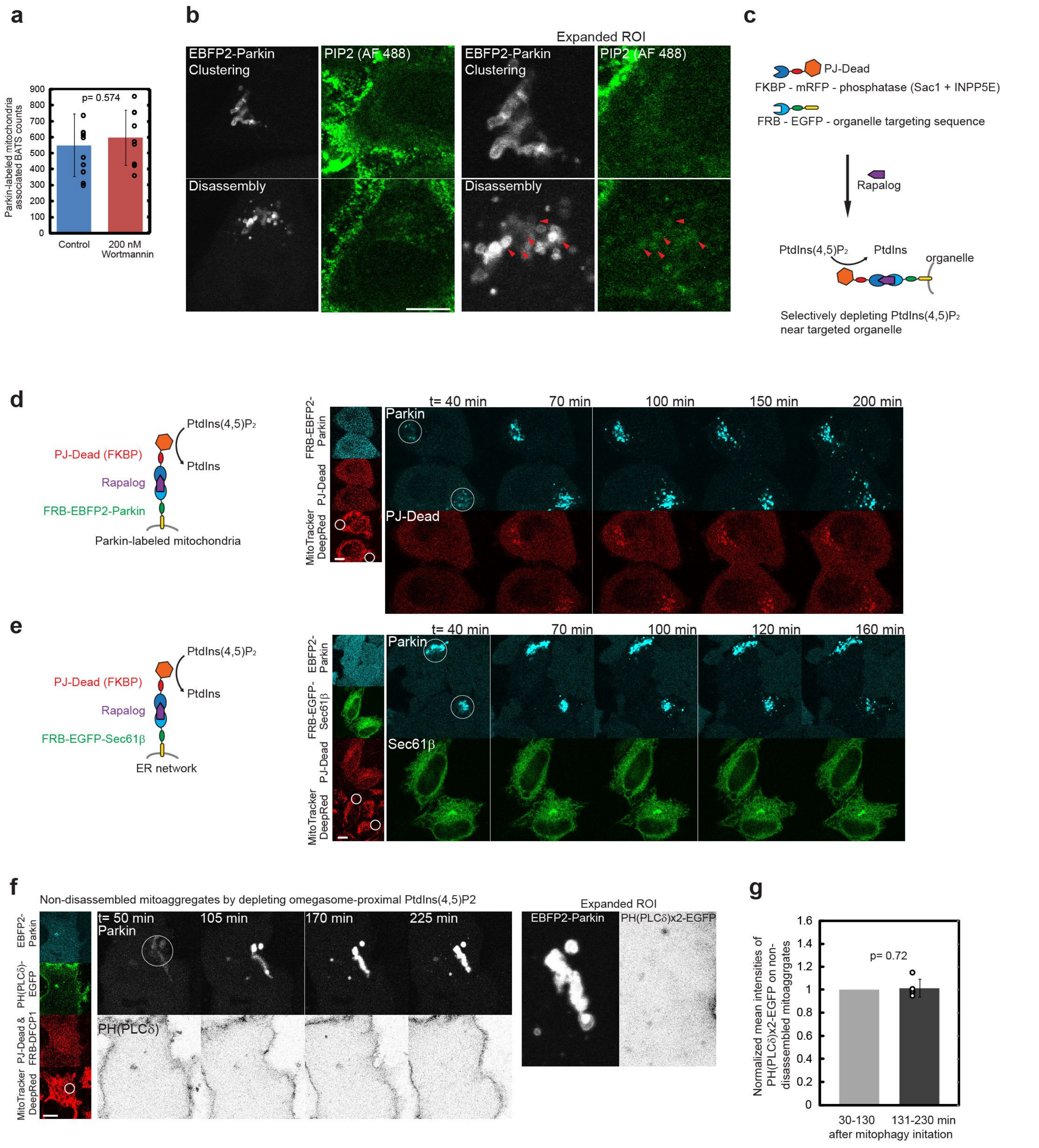

Supplementary Figure 11. Mapping the mitoaggregate disassembly related PtdIns(4,5)$_2$ pool using organelle targeting PJ-Dead (**a**) Quantifying the the effect of wortmannin, a PtdIns 3-kinase inhibitor, on the appearance of BATS-labeled structures near Parkin-labeled mitochondria. Mitoaggregate-associated BATS counts were not affected by PtdIns(3)P depletion. Wortmannin-treated HeLa cells co-expressing EBFP2-Parkin, PJ-Dead, FRB-DFCP1, and hrGFP-BATS were stained with MitoTracker DeepRed FM followed by 635 nm illumination to locally initiate mitophagy. Total BATS counts near Parkin-labeled mitochondria were calculated from 200 min time-lapse images with 2 min interval (mean±S.D.; control n=11, wortmannin treated n=9 biologically independent samples; p-value as evaluated using two-tailed unpaired Student's t-test). (**b**) Immunofluorescence imaging of PIP2 during mitophagy. Intracellular PIP2 was observed near disassembled mitoaggregate. (**c**) Diagram illustrating the principle underlying rapalog-induced recruitment of PJ-Dead to targeted cellular locations, leading to the local depletion of PtdIns(4,5)P$_2$. (**d-e**) Rapalog-treated HeLa cells co-expressing FRB-EBFP2-Parkin and PJ-Dead (in **d**) or EBFP2-Parkin, PJ-Dead, and FRB-EGFP-Sec61β (in **e**) were stained with MitoTracker DeepRed FM followed by 635 nm illumination (white circles) to locally initiate mitophagy. Rapalog-induced PJ-Dead recruitment to damaged mitochondria or ER networks did not affect mitoaggregate disassembly. (**f**) PtdIns(4,5)P$_2$ levels, indicated by the intensity of PH(PLCδ)x2-EGFP, did not increased on non-disassembled mitoaggregates. Rapalog-treated HeLa cells co-expressing EBFP2-Parkin, PJ-Dead, FRB-DFCP1, and PH(PLCδ)x2-EGFP were stained with MitoTracker DeepRed FM followed by 635 nm illumination to locally initiate mitophagy. (**g**) Mean intensities of PH(PLCδ)x2-EGFP on non-disassembled mitoaggregates induced by omegasome-targeting PJ-Dead. (mean±S.D.; n=5 biologically independent samples; p-value as evaluated using two-tailed unpaired Student's t-test). Scale bar: 10 μm

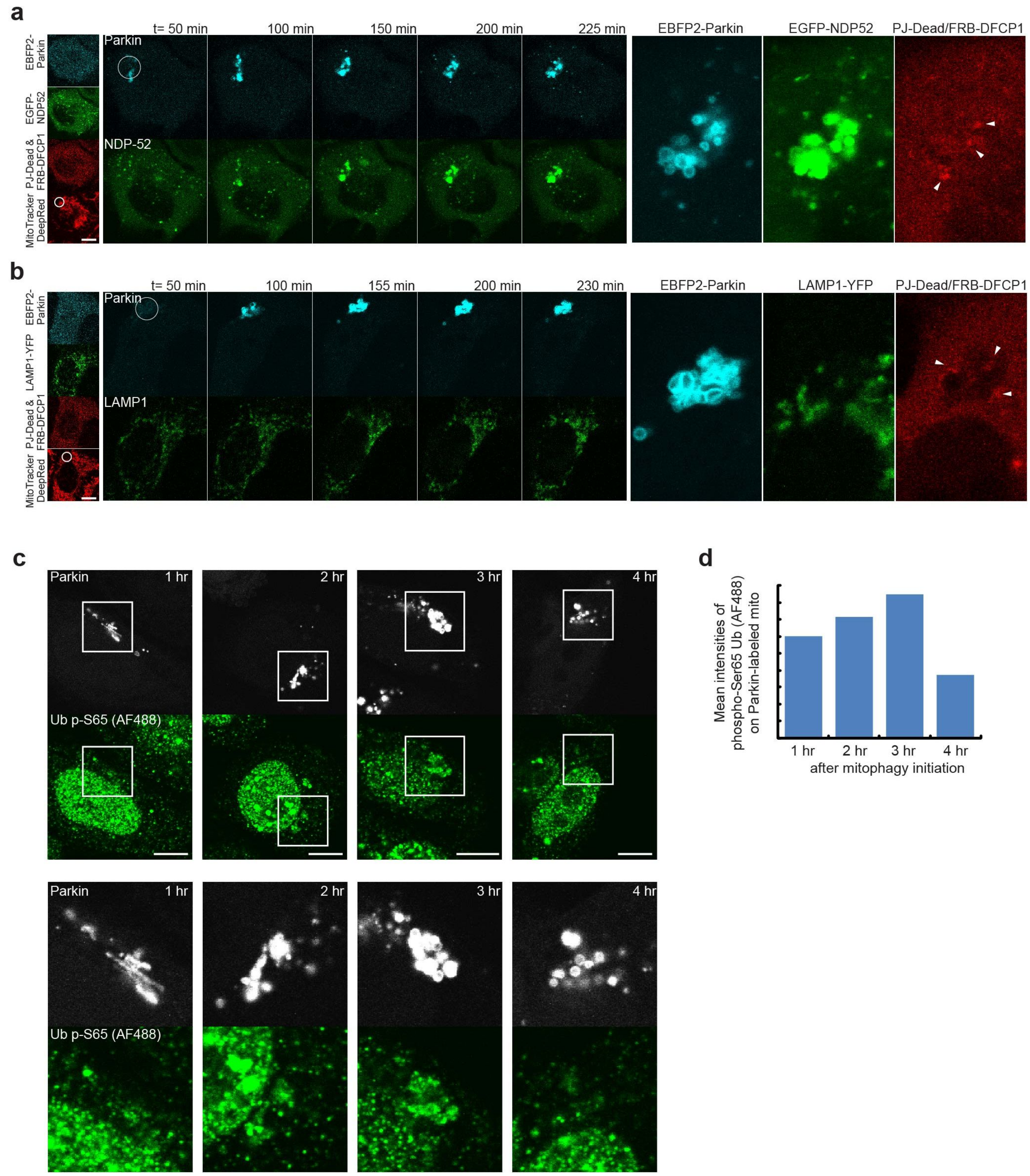

Supplementary Figure 12. Properties of non-disassembled mitoaggregates. (**a**-**b**) Rapalog-treated HeLa cells co-expressing EBFP2-Parkin, PJ-Dead, FRB-DFCP1, and EGFP-NDP52 (in **a**) or LAMP1-YFP (in **b**) were stained with MitoTracker DeepRed FM followed by 635 nm illumination to locally initiate mitophagy. Non-disassembled mitoaggregates, induced by omegasome-proximal PtdIns(4,5)P_2 depletion, have NDP52 labeling (as in **a**), but could not be delivered into lysosomes (as in **b**). Rapalog-induced PJ-Dead recruitments to omegasomes were indicated by white arrows shown in right magnified views. (**c**) Immunofluorescence imaging of phosphor-Ser65 Ub during mitophagy. Levels of phosphor-Ser65 Ub on damaged mitochondria increased during mitochondrial clustering and decreased following mitoaggregate disassembly (4 hr after mitophagy initiation). Magnified views of the white square regions were shown in lower panel. (**d**) Intensities of immunofluorescence signals of phosphor-Ser Ub on Parkin-labeled mitochondria as shown in **c**. Scale bar: 10 μm

Hsieh et al. Supplementary information. Supplementary Table 1

Primers for cloning		
Primer name	Sequence	Construct
5' NheI-EB3 primer	TCCGCTAGCGATGGCCGTCAATGTGTACTCCA	EB3-EGFP
3' EB3-AgeI primer	GGCGACCGGTAGGTACTCGTCCTGGTCTTCTTGTTGAT	
5' NheI-vimentin primer	CCGCTAGCGATGTCCACCAGGTCCGTGTCC	EGFP-vimentin
3' vimentin-AgeI primer	CGACCGGTAGTTCAAGGTCATCGTGATGCTGAGA	
5' EcoRI-p62 primer	TTCGAATTCTATGGCGTCGCTCACCGTGAA	EGFP-p62
3' p62-XmaI primer	GATCCCGGGTCACAACGGCGGGGGATG	
5' NheI-PH(PLCδ) primer	TCCGCTAGCGATGGACTCGGGCCGGG	PH(PLCδ)x2-EGFP
3' PH(PLCδ)-linker-SalI primer	ACCGTCGACTCCAGAGCCACCGCCACCCTTCAGGAAGTTCTGCAGCTC	
5' SalI-linker-PH(PLCδ) primer	GGAGTCGACGGTGGTGGGTCCGGAGGAATGGACTCGGGCCGGG	
3' PH(PLCδ)-AgeI primer	GCGACCGGTAGCTTCAGGAAGTTCTGCAGCTCCT	
5' EcoRI- DFCP1 primer	TTCGAATTCTATGAGTGCCCAGACTTCCCCA	EGFP-DFCP1
3' DFCP1-BamHI primer	GGTGGATCCTTAAAGGTCACCGGGCTTTTTATTG	
5' EcoRI-Sec61β primer	TTCGAATTCTATGCCTGGTCCGACCCC	EGFP-Sec61β
3' Sec61β-SalI primer	ACCGTCGACCTACGAACGAGTGTACTTGCCCC	
5' NheI-TPC2 primer	TCCGCTAGCGATGGCGGAACCCCAGGC	TPC2-TagRFP
3' TPC2-AgeI primer	GCGACCGGTAGCCTGCACAGCCACAGGTGC	
